# Chitosan Hydrochloride Decreases *Fusarium graminearum* Growth and Virulence and Boosts Growth, Development and Systemic Acquired Resistance in Two Durum Wheat Genotypes

**DOI:** 10.3390/molecules25204752

**Published:** 2020-10-16

**Authors:** Sara Francesconi, Barbara Steiner, Hermann Buerstmayr, Marc Lemmens, Michael Sulyok, Giorgio Mariano Balestra

**Affiliations:** 1 Department of Agriculture and Forest Sciences (DAFNE), University of Tuscia, Via San Camillo de Lellis, snc, 01100 Viterbo, Italy; 2 Department of Agrobiotechnology Tulln (IFA-Tulln), University of Natural Resources and Life Sciences Vienna (BOKU), Konrad Lorenz Straße 20, A-3430 Tulln an der Donau, Austria; barbara.steiner@boku.ac.at (B.S.); hermann.buerstmayr@boku.ac.at (H.B.); marc.lemmens@boku.ac.at (M.L.); Michael.sulyok@boku.ac.at (M.S.)

**Keywords:** chitosan, fusarium head blight, durum wheat, plant defense, systemic acquired resistance, fungicides

## Abstract

Fusarium head blight (FHB) is a devastating disease for cereals. FHB is managed by fungicides at anthesis, but their efficacy is variable. Conventional fungicides accumulate in the soil and are dangerous for animal and human health. This study assayed the antifungal ability of chitosan hydrochloride against *Fusarium graminearum*. Chitosan reduced *F. graminearum* growth and downregulated the transcript of the major genes involved in the cell growth, respiration, virulence, and trichothecenes biosynthesis. Chitosan promoted the germination rate, the root and coleoptile development, and the nitrogen balance index in two durum wheat genotypes, Marco Aurelio (FHB-susceptible) and DBC480 (FHB-resistant). Chitosan reduced FHB severity when applied on spikes or on the flag leaves. FHB severity in DBC480 was of 6% at 21 dpi after chitosan treatments compared to *F. graminearum* inoculated control (20%). The elicitor-like property of chitosan was confirmed by the up-regulation of *TaPAL*, *TaPR1* and *TaPR2* (around 3-fold). Chitosan decreased the fungal spread and mycotoxins accumulation. This study demonstrated that the non-toxic chitosan is a powerful molecule with the potential to replace the conventional fungicides. The combination of a moderately resistant genotype (DBC480) with a sustainable compound (chitosan) will open new frontiers for the reduction of conventional compounds in agriculture.

## 1. Introduction

Wheat is one of the most cultivated crops worldwide and its annual production is estimated to be more than 700 million tons. Wheat accounts for more than 20% of total human food calories, it is extensively grown on 17% of all crop areas, and it is the staple food for 40% of the world’s population, comprising Europe, North America, and the western and northern parts of Asia [1,2]. Modern wheat cultivars refer to two species, the hexaploid bread wheat, *Triticum aestivum* L. and the tetraploid durum-type, *T. turgidum* subsp. *durum* (Desfontaines) Husnache [3,4]. Particularly, durum wheat is the most widespread crop in the Mediterranean area and around 77% of the Italian production of durum wheat comes from the Central and Southern regions, mainly employed for the production of pasta [5].

Fusarium head blight (FHB) is caused by the *Fusarium* species and is considered as the most devastating wheat disease due to the lack of resistant cultivars, significant yield loss and grain quality reduction, and health threat of wheat food and feed contaminated with mycotoxins produced by the causal agents [6,7]. FHB epidemics have occurred regularly in the wheat-growing regions such as Asia, North and South America, and Europe [8].

*F. graminearum* Schwabe is the most aggressive FHB causal agents. *Fusarium* spp. are fungal hemi-biotrophic pathogens and in field conditions the inoculum arises primarily from plant residues and soils and its dissemination is mainly promoted by water splash and wind [9,10]. Anthesis is the most sensitive phenological stage to the infection and, with a warm and humid climate, FHB develops rapidly within 3–6 days after the infection. On wheat spikelets, airborne spores germinate and enter the plants via degenerated anther tissues or minute natural openings under the lemma. Further, the growth of the fungus occurs between the cells and it passes from the xylem and the pits and colonizes the tissue, forming necrosis. This leads to the water soaking tissues causing the production of shriveled kernels and premature bleaching that affects photosynthesis [11,12,13,14]. After the infection, genes for deoxynivalenol (DON) biosynthesis are expressed by the fungus, facilitating the spreading of the pathogen from spikelets to rachis [15,16].

Fungicide application is one of the strategies available for the management of FHB and DON [17]. Triazole fungicides containing tebuconazole are currently the most effective [18,19] and it is reported that the application of tebuconazole decreased the levels of DON in artificially inoculated plants [18,19,20], but also conflicting results have also been obtained [21]. Another class of fungicides particularly in use in Italy for FHB control are strobilurins, comprising azoxystrobin, mixed with triazole compounds. Nevertheless, strobilurin fungicides are generally ineffective in controlling FHB, and their application tends to increase DON content as compared to nontreated control [22]. The efficacy of conventional chemical fungicides for FHB is variable and many researchers reported less than 50 to 60% of control [19,23]. Moreover, from recent studies, the occurrence of fungicide- resistant *Fusarium* strains, due to the over-use of the same types of compounds, has been reported. Notably, a *F. graminearum* isolate that is resistant to tebuconazole has been discovered in the USA [24] as well as some strains that are resistant to benzimidazole-based fungicides in China [25]. Fungicides not only affect the target organism, but the whole environment, including atmosphere, soil, groundwater, and surface water by flow, leaching, and pulverization processes, thus contaminating the ecosystem [26,27]. The presence of fungicides in the ecosystems have adverse effects that vary with the contaminant concentration, amount, and exposure time [28,29]. As a consequence, they may present detrimental effects to humans and can cause severe health problems such as cancer, infertility, malformation, and chromosomal changes as a consequence of oxidative stress [30]. For those reasons, these contaminants should be removed from the environment, especially from water. In fact, severe restrictions have been posed on the use of compounds whose characteristics are persistence, bioaccumulation, toxicity, and long-range environmental transport [31,32]. To respond to the actual increasing demand of food and the concomitant need to reduce the use of conventional pesticides, the scientific research needs to focus on sustainable agricultural production systems [33,34]. In this scenario, natural-derived compounds represent a novel strategy for the replacement of conventional agrochemicals [35,36,37].

Chitosan has been one of the most interesting biopolymers due to its biocompatibility, antioxidant, anticancer, biodegradability, antimicrobial, and non-toxic properties as well as being an economical material, produced from waste resources such as seafood shells [38,39]. Chitosan is a derivate from chitin, and, besides agriculture, it has a large set of applications, such as in the food, cosmetic, textile, and biomedical industries [40,41].

In plants, chitosan is largely used to mimic biotic and abiotic stresses. The first study of using chitosan as an antimicrobial agent in plants was reported by Allan and Hadwiger (1979) [42], where they demonstrated the fungicidal effects of chitosan on different cell wall compositions of fungi. The improvement of the defense system after applying chitin and chitosan has been observed in both monocotyledons and dicotyledons [43]. Chitosan has been a bio-fungicide, bio-bactericide, and bio-virucide, which boosts plant defense systems against the pathogen, thus inducing the natural defense system of plants, fruits, and vegetables [44,45,46]. It was also found to be involved in altering the growth of fungi and to reduce toxin production [47]. Moreover, chitosan use is recommended to enhance the photosynthetic activity, vegetative growth, antioxidant activities, fruit quality attributes, and overall growth and yield of the crop [39]. This body of research suggests that chitosan can be used as biostimulant of the plant immune system to challenge diverse hostile environments. In addition, many research studies stated that pathogenesis-related (PR) genes and several defense-related enzymes such as phenylalanine ammonia-lyase (PAL) are induced by chitosan [39,48,49,50].

In this context, the focus of the present research study was to test the inhibitory effect of chitosan hydrochloride against *F. graminearum* by in vitro and in vivo assays. Four different in vitro assays were performed to study the mode of action of chitosan against *F. graminearum*, while two durum wheat genotypes (Marco Aurelio, FHB susceptible, and DBC480, FHB resistant) were employed as plant host during in vivo experiments.

## 2. Results

### 2.1. In Vitro Antifungal Activity of Chitosan Hydrochloride

**Figure 1:**
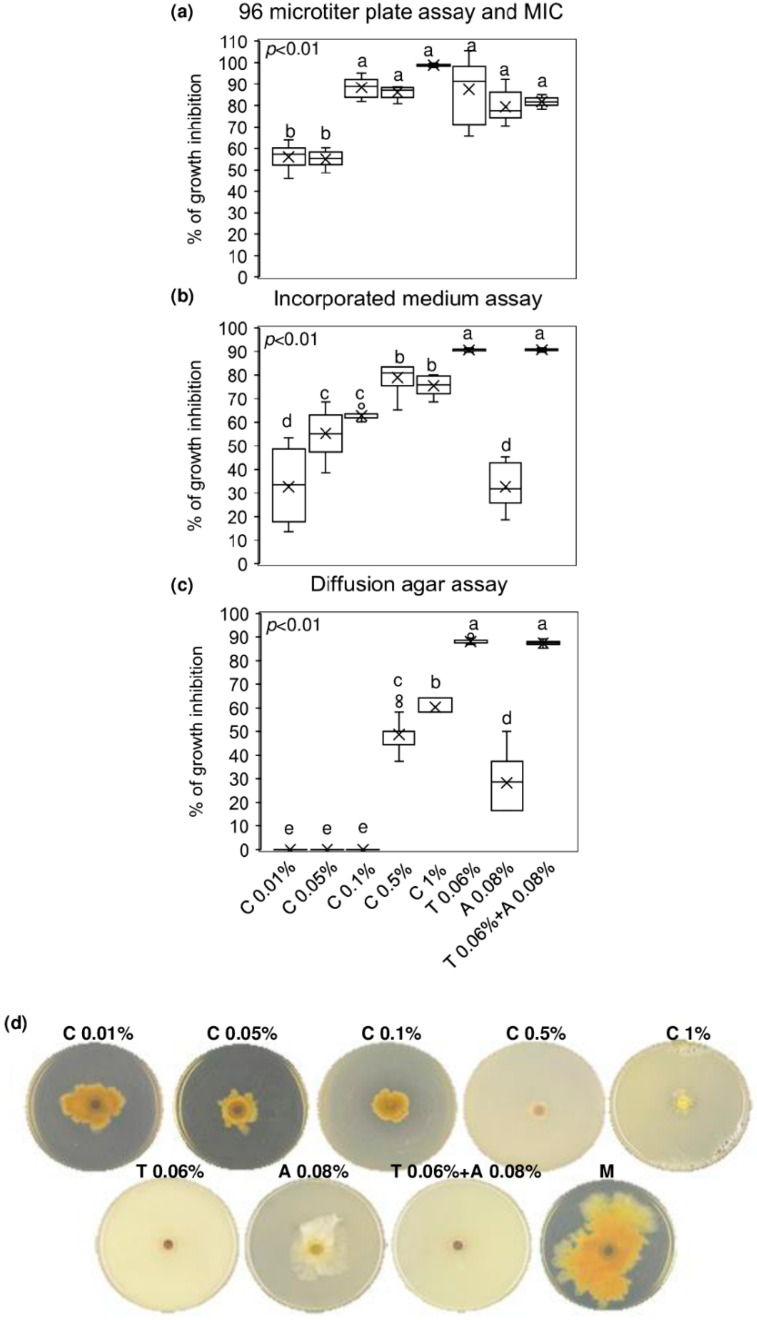
In vitro antifungal assays. Boxplots of (**a**) 96 microtiter plates assay and determination of the minimum inhibitory concentration (MIC); (**b)** incorporated medium assay; (**c**) diffusion agar assay. (**d**) *F. graminearum* growth on Potato Dextrose Agar (PDA) incorporated with the tested compounds. Chitosan hydrochloride (C) was tested at 0.01–0.05–0.1–0.5 and 1%, tebuconazole (T) and azoxystrobin (A) were used as control fungicides at the recommended field doses of 0.06% and 0.08%, respectively, and were assayed alone or mixed (T + A). M represents the Mock control. Results are expressed as % of growth inhibition. The data represent averages and standard errors from three independent experiments, each one consisting of four replicates for each substance and concentration tested. Different letters (a, b, c, d, e) into the subfigures refer to the statistical analysis performed using one-way analysis of variance (ANOVA) with the Tukey test at a confidence level of 0.99 and *p* < 0.01.

The minimum inhibitory concentration (MIC) value of chitosan hydrochloride was established by a 96 microtiter plates assay and the concentration at 1% resulted to inhibit completely *F. graminearum* growth. Notably, lower concentrations of chitosan also resulted in being effective in controlling *F. graminearum* growth, since the % of inhibition ranged from 53 to 87% after treating the conidia of *F*. *graminearum* with chitosan at 0.01–0.05–0.1 and 0.5%. Importantly, chitosan at 0.1–0.5 and 1% demonstrated the ability to inhibit *F. graminearum* as much as the reference fungicides (tebuconazole, azoxystrobin, and the mixture of tebuconazole and azoxystrobin) used at the recommended field doses (0.06% for tebuconazole and 0.08% for azoxystrobin) (Figure 1a). After seven days of culturing *F. graminearum* in chitosan-incorporated PDA, none of the chitosan concentrations tested was as effective as tebuconazole and the mixture of tebuconazole and azoxystrobin in limiting *F. graminearum* radial growth. However, chitosan at 0.05–0.1–0.5 and 1% showed remarkable fungal inhibition (from 55 to 75%), resulting in being more effective than azoxystrobin, which reduced the *F. graminearum* growth of 32% (Figure 1b). Figure 1d evidences the radial growth of *F. graminearum* after seven days of incubation on incorporated PDA with chitosan at different concentrations, reference fungicides, and mock control. Finally, Figure 1c shows that only chitosan at 0.5 and 1% inhibited *F. graminearum* by agar diffusion (48 and 60% of inhibition, respectively) more efficiently than azoxystrobin but not as much as tebuconazole and the mixture of tebuconazole and azoxystrobin.

**Figure 2:**
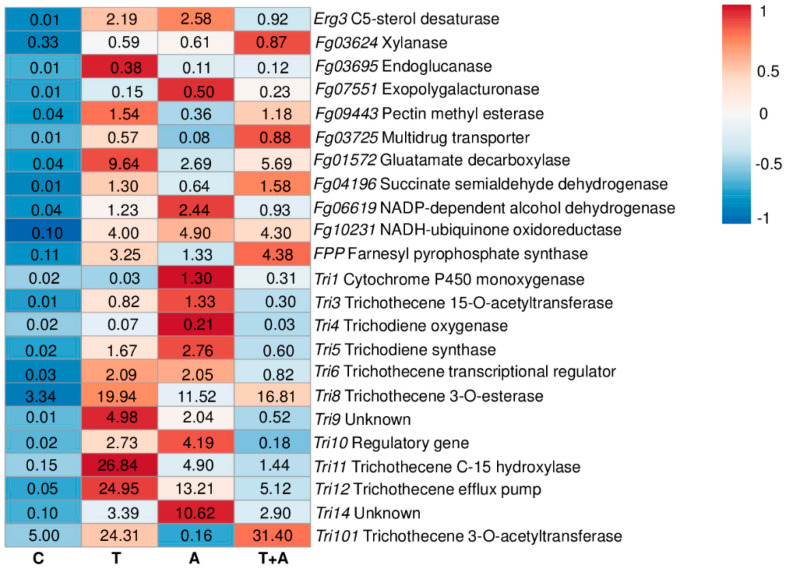
Heatmap and relative expression levels of the selected *F. graminearum* genes. The relative expression levels are referred to the different compounds’ treatments (Chitosan hydrochloride C, Tebuconazole T, Azoxystrobin A, Tebuconazole+Azoxystrobin T + A). Relative expression values were obtained by using the equation 2^−ΔΔCq^ with *TEF*, *TUB*, and *GAPDH* as reference genes and mock treatment used to normalize the relative expression levels. The heatmap was constructed with the z-score by analyzing data with ClustVis software (Tartu, Estonia).

In the present study, we also evaluated the relative transcripts level of some major *F. graminearum* genes responsible for redox balance, cell respiration, nutrient transport, cell wall degrading enzymes, ergosterol biosynthesis, and trichothecenes production, in order to evaluate the mode of action of chitosan hydrochloride compared to the reference fungicides on the physiology and virulence of *F. graminearum*. Figure S1 shows RNA integrity (a) and *TEF* amplification (b) from *F. graminearum,* demonstrating that the cDNA was successfully synthetized. The reaction efficiency (E) and R^2^ values ranged from 0.92 to 1.20 and from 0.97 to 0.99, respectively, from each primer pair. Figure 2 shows the heatmap and the relative expression values for each gene and compound treatments, while Table S1 also provides the standard errors and statistical analysis according to the Tukey test computed at 0.99 confidence level.

Chitosan hydrochloride only upregulated *Tri8* (3.34-fold) and *Tri101* (5.00-fold), while the rest of the genes were strongly downregulated. Tebuconazole demonstrated a remarkable induction of the *Tri* pathway, among which *Tri8*, *Tri11*, *Tri12,* and *Tri101* showed relative expression values of 19.94-, 26.84-, 24.95-, and 24.31-fold. Azoxystrobin also promoted the upregulation of the *Tri* genes, even if not as much as tebuconazole. The mixture of tebuconazole and azoxystrobin drastically reduced the induction of the *Tri* pathway, with the exception of *Tri8* and *Tri101* (16.81- and 31.40-fold, respectively). On the other hand, the genes involved in redox balance, cell respiration, nutrient up-take, cell wall degrading enzymes, and ergosterol biosynthesis were mostly down-regulated after each type of treatment, with the exception of *Erg3*, *Fg09943*, *Fg01572*, *Fg04196*, *Fg06619,* and *Fg10231* after tebuconazole treatment, *Erg3*, *Fg01572*, *Fg06619,* and *Fg10231* after azoxystrobin treatment, *Fg09443*, *Fg01572*, *Fg04196,* and *Fg10231* after the mixture treatment (Figure 2 and Table S1)

### 2.2. Biostimulant Effects of Chitosan Hydrochloride on Durum Wheat

**Figure 3:**
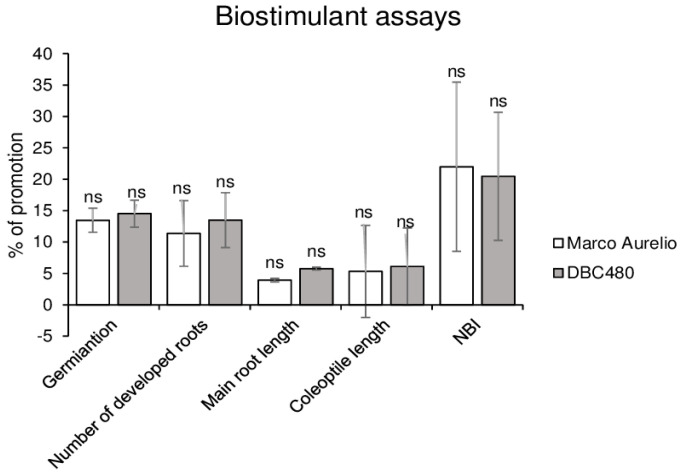
In vivo biostimulant assays. The biostimulant effect of chitosan hydrochloride at 0.5% was tested on Marco Aurelio and DBC480 by evaluating the kernels germination, number of developed roots, main root length, coleoptile length, and Nitrogen Balance Index (NBI). Results are expressed as % of growth inhibition. The data represent averages and standard errors from three independent experiments organized as a complete randomized block, each one consisting of 20 plants for the experimental group (wheat genotype × compound). The statistical analysis was performed using one-way analysis of variance (ANOVA); “ns” refers to “not significant”.

Kernels of Marco Aurelio and DBC480 were coated with chitosan hydrochloride at 0.5% or water in order to evaluate their effects on durum plants. As shown in Figure 3, chitosan promoted the germination rate (14%), the number of developed roots (12%), the length of the main root (4%), and coleoptile (6%) and the chlorophyll and flavonoids ratio expressed as NBI values (21%) both in Marco Aurelio and DBC480 seedlings. No significant differences were observed in the growth between the two durum wheat genotypes after the chitosan coating.

Figure S2 shows the growth of the durum seedling at 14 days after the coating with the different compounds.

### 2.3. In Vivo Antifungal Activity of Chitosan Hydrochloride and Its Effectiveness on Systemic Acquired Resistance (SAR) Induction in Durum Wheat

**Figure 4:**
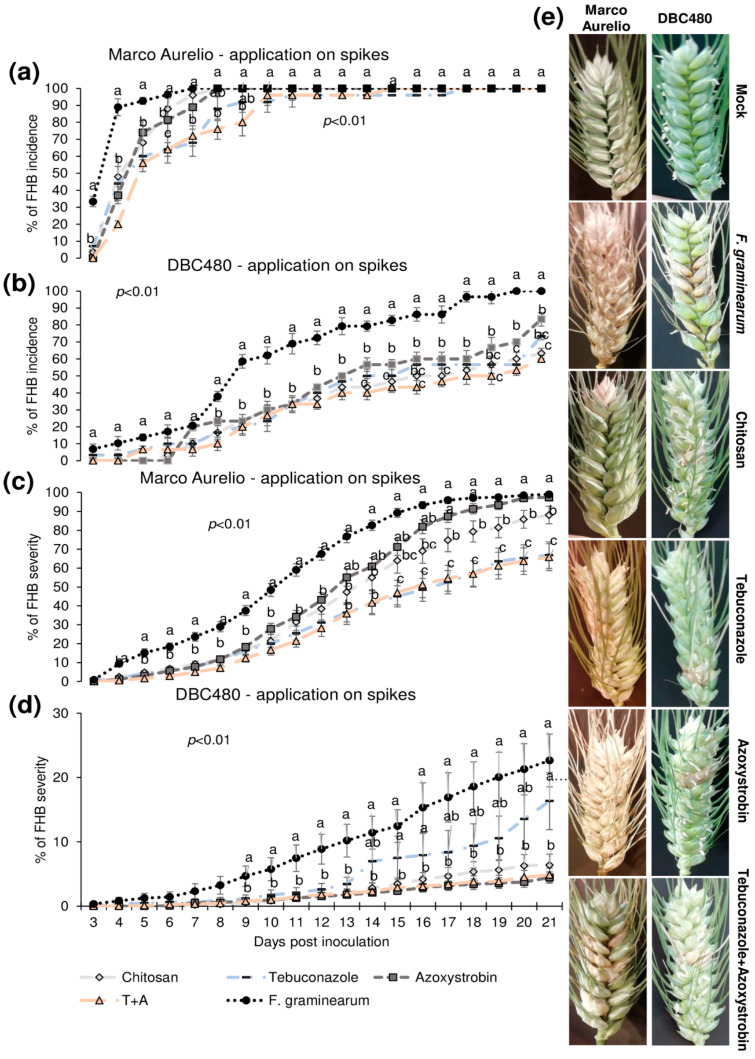
Fusarium head blight (FHB) incidence and severity (%) in (**a**,**c**) Marco Aurelio and (**b**,**d**) DBC480 from 3 to 21 days post inoculation (dpi) and (**e**) FHB symptoms at 21 dpi for plants treated 48 h before the *F. graminearum* inoculation at the spike level with chitosan at 0.5%, tebuconazole at 0.06%, azoxystrobin at 0.08%, the mixture of tebuconazole at 0.06%, and azoxystrobin at 0.08%. Merely *F. graminearum* inoculated and mock plants were used as controls. The data represent averages and standard errors from three independent replicates with at least 10 spikes for each experimental group (wheat genotype × compound). Different letters (a, b, c) into the subfigures refer to the statistical analysis performed using one-way analysis of variance (ANOVA) with the Tukey test at a confidence level of 0.99 and *p* < 0.01.

Plants of Marco Aurelio and DBC480 were preventively treated with chitosan hydrochloride at 0.5% or the reference fungicides at the previously cited concentrations 48 h before the inoculation. The compounds were applied directly on the spikes or on the flag leaves. Figure 4 illustrates the FHB incidence and severity (%) from 3 to 21 dpi after applying the compounds on the spikes. Chitosan reduced the FHB incidence as much as the reference fungicides from 3 to 5 dpi in Marco Aurelio (a) (FHB incidence was of 75%, while it was of 92% in *F. graminearum* inoculated control plants), at 6 dpi its effect was similar to azoxystrobin, while from 7 to 9 dpi, only tebuconazole and the mixture of the reference fungicides reduced the FHB incidence. From 10 to 21 dpi, none of the compounds reduced the FHB incidence. In DBC480 plants (b), chitosan reduced the FHB incidence as much as the reference fungicides from 3 to 13 dpi (FHB incidence was of 50%, while it was of 80% in *F. graminearum* inoculated control plants), while from 14 to 21 dpi showed a similar effect in reducing FHB incidence to the mixture of the reference fungicides, demonstrating a higher efficacy than tebuconazole and azoxystrobin applied alone. At 21 dpi, chitosan and the mixture of fungicides-treated plants showed an FHB incidence of 60%, while it was of 69%, 80%, and 100% in tebuconazole, azoxystrobin, and *F. graminearum* control plants, respectively. Chitosan decreased FHB severity in Marco Aurelio (c) as much as the reference fungicides from 4 to 16 dpi, and from 17 to 21 dpi chitosan was more effective than azoxystrobin but not as much as tebuconazole and the mixture of tebuconazole and azoxystrobin. At 21 dpi, *F. graminearum* inoculated control and azoxystrobin-treated plants showed 100% of FHB severity, 85% after chitosan treatment, and 60% after tebuconazole and the mixture of the reference fungicides treatments. On DBC480 (d), chitosan reduced the FHB severity as much as the reference fungicides from 9 to 21 dpi (6% of FHB severity). FHB severity in *F. graminearum* inoculated control plants was 20% and in azoxystrobin-treated plants was 15%. Figure 4e shows FHB symptoms at 21 dpi on Marco Aurelio and DBC480 subjected to the different compounds’ treatments at the spike level and mock plants, and no FHB symptoms were recorded.

**Figure 5:**
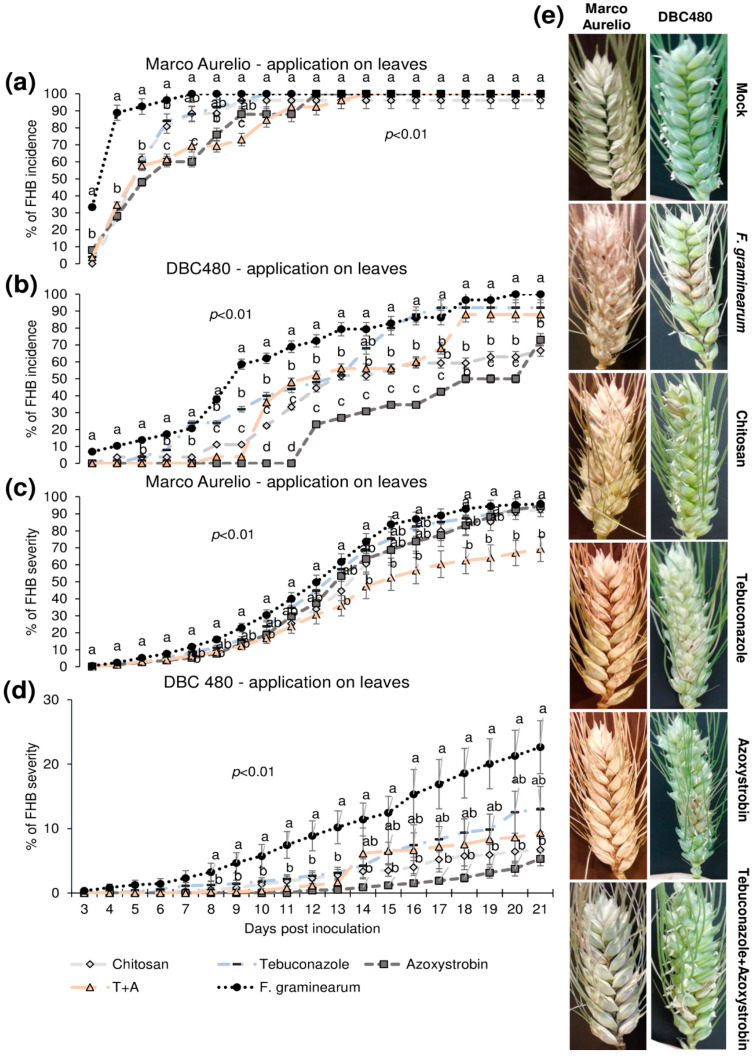
Fusarium head blight (FHB) incidence and severity (%) in (**a**,**c**) Marco Aurelio and (**b**,**d**) DBC480 from 3 to 21 days post inoculation (dpi) and (**e**) FHB symptoms at 21 dpi for plants treated 48 h before the *F graminearum* inoculation at the flag leaf level with chitosan at 0.5%, tebuconazole at 0.06%, azoxystrobin at 0.08%, the mixture of tebuconazole at 0.06%, and azoxystrobin at 0.08%. Merely *F. graminearum* inoculated and mock plants were used as controls. The data represent averages and standard errors from three independent replicates with at least 10 spikes for each experimental group (wheat genotype × compound). Different letters (a, b, c) into the subfigures refer to the statistical analysis performed using one-way analysis of variance (ANOVA) with the Tukey test at a confidence level of 0.99 and *p* < 0.01.

Figure 5 shows the FHB incidence and severity from 3 to 21 dpi after applying the compounds on the flag leaves. In Marco Aurelio plants (a), chitosan reduced the FHB incidence as much as the reference fungicides from 3 to 5 dpi (FHB incidence of 60% in chitosan treated plants, while it was 90% in the *F. graminearum* inoculated control plants without pre-treatment), while at 6 dpi the FHB incidence of chitosan and tebuconazole treated plants was reduced but not as much as in azoxystrobin and the mixture of tebuconazole and azoxystrobin treated plants. From 7 to 21 dpi, no FHB reduction was observed in chitosan and tebuconazole treated plants, while azoxystrobin and the mixture of the reference fungicides reduced the FHB severity from 6 to 8 dpi and from 6 to 9 dpi, respectively. From 10 to 21 dpi, the FHB severity was of 100% in all the experimental groups. In DBC480 plants (b), chitosan reduced the FHB incidence from 3 to 6 dpi as much as the reference fungicides; from 7 to 9 dpi chitosan was more effective than tebuconazole and from 10 to 11 dpi and from 18 to 21 dpi chitosan significantly reduced the FHB incidence more than tebuconazole and the mixture of the reference fungicides (FHB incidence of 70% in chitosan and 90% in tebuconazole and the mixture of the reference fungicides treated plants at 21 dpi, respectively). Interestingly, the azoxystrobin compound was the most effective in reducing FHB incidence from 10 to 20 dpi, while the FHB incidence of azoxystrobin treated plants at 21 dpi was similar to that observed in chitosan treated plants. FHB severity in Marco Aurelio plants (c) was not significantly reduced for the entire infection trial, with the exception of the mixture of the reference fungicides, which reduced FHB severity from 15 to 21 dpi (FHB severity of 65%, while it was of 90% in *F. graminearum* inoculated control plants). In DBC480 plants (d), all the treatments reduced the FHB severity from 8 to 13 dpi, while from 14 to 21 dpi only chitosan and azoxystrobin significantly reduced the FHB severity compared to *F. graminearum* inoculated control plants (FHB severity was of 6%, while it was of 21% in *F. graminearum* inoculated control plants). Figure 5e shows FHB symptoms at 21 dpi on Marco Aurelio and DBC480 subjected to the different compounds’ treatments at the flag leaf level and mock plants, where no FHB symptoms were recorded.

**Figure 6:**
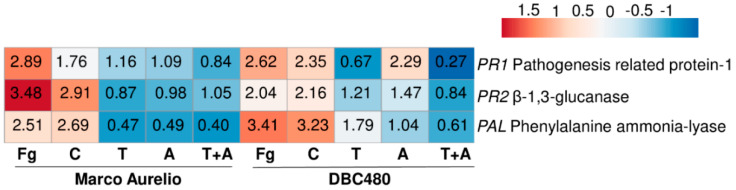
Heatmap and relative expression levels of the selected durum wheat genes. The relative expression levels are referred to the different compounds’ pre-treatments (Chitosan C, Tebuconazole T, Azoxystrobin A, Tebuconazole + Azoxystrobin T + A) applied on the flag leaf and to the two wheat genotypes Marco Aurelio and DBC480. Fg represents the *F. graminearum* inoculated control. Relative expression values were obtained by using the equation 2^−ΔΔCq^ with *TaFNR*, *TaTUB*, and *TaACT* as reference genes and mock treatment used to normalize the relative expression levels. The heatmap was constructed with the z-score by analyzing data with ClustVis software (Tartu, Estonia).

From Marco Aurelio and DBC480 plants treated at the flag leaf level, the relative transcript levels of *TaPR1*, *TaPR2,* and *TaPAL* were evaluated, in order to assess the putative induction of SAR by the different plant treatments. Figure S3 shows RNA integrity (a) and *TaACT* amplification (b) from Marco Aurelio and DBC480, demonstrating that the cDNA was successfully synthetized. E and R^2^ values ranged from 0.97 to 1.11 and from 0.97 to 0.99, respectively, from each primer pair. Figure 6 shows the heatmap and the relative expression values for each gene and compound treatments, while Table S2 also provides the standard errors and statistical analysis according to the Tukey test computed at 0.99 confidence level.

*TaPAL* was up-regulated after *F. graminearum* inoculation (2.89- and 2.62-fold in Marco Aurelio and DBC480, respectively) and chitosan treatments (1.76- and 2.35-fold in Marco Aurelio and DBC480, respectively). Notably, the upregulation of *TaPAL* in DBC480 plants after the chitosan treatment was significantly higher than in Marco Aurelio plants. *TaPAL* was also upregulated in DBC480 plants treated with azoxystrobin (2.29-fold) but basal-regulated in Marco Aurelio (1.09-fold). *TaPAL* was also basal-regulated after tebuconazole treatment in Marco Aurelio and slightly downregulated in Marco Aurelio plants treated with the mixture of the reference fungicides and in DBC480 plants treated with tebuconazole and the mixture of the reference fungicides. *TaPR1* was promptly induced (3.48-fold) *in F. graminearum* inoculated Marco Aurelio plants and it was also induced in *F. graminearum* inoculated DBC480 (2.04-fold) and chitosan treated plants (2.91- and 2.16-fold in Marco Aurelio and DBC480, respectively). Tebuconazole, azoxystrobin, and the mixture of the two reference fungicides basal-regulated the genes in both Marco Aurelio and DBC480, with the exception of azoxystrobin in DBC480 plants, that slightly upregulated *TaPR1* (1.47-fold). *TaPR2* was promptly induced in *F. graminearum* inoculated and chitosan treated DBC480 plants (3.41- and 3.23-fold). The *F. graminearum* artificial inoculation, chitosan treatment in Marco Aurelio, and tebuconazole application in DBC480 also induced *TaPR2* (2.51-, 2.69-, and 1.79-fold, respectively), while the gene resulted basal-regulated after the application of azoxystrobin in DBC480 plants anddown-regulated after tebuconazole, azoxystrobin and the mixture of the reference fungicides application in Marco Aurelio (Figure 6 and Table S2).

### 2.4. Chitosan Hydrochloride Effectiveness on Maintaining Grain Yield, Containing F. graminearum Biomass Spread and FHB-Associated Compounds Accumulation

**Figure 7:**
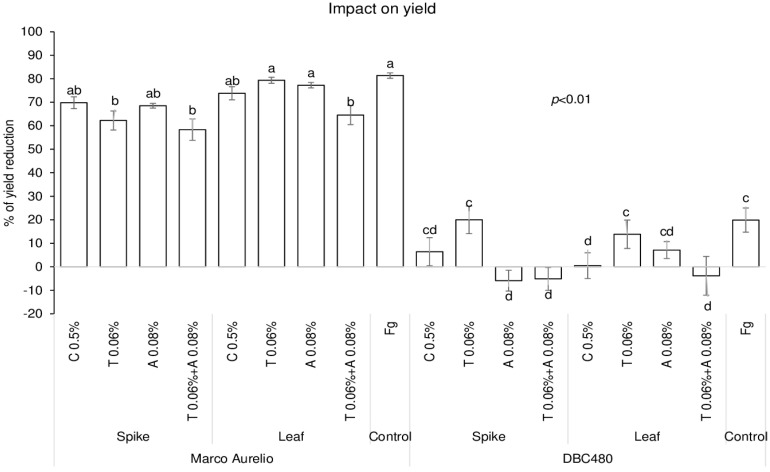
Percent yield reduction in Marco Aurelio and DBC480 due to *F. graminearum* infection after treating the plants at the spike or flag leaf level with Chitosan at 0.5% (C 0.5%), tebuconazole at 0.06% (T 0.06%), azoxystrobin at 0.08% (A 0.08%), the mixture of tebuconazole at 0.06% and azoxystrobin at 0.08% (T 0.06% + A 0.08%) prior to *F. graminearum* inoculation; *F. graminearum* inoculated spikes served as controls (Fg). The data represent averages and standard errors from three independent replicates with at least 10 plants for each experimental group (wheat genotype × compound × mode of application). Different letters (a, b, c, d) refer to the statistical analysis performed using three-way analysis of variance (ANOVA) with the Tukey test at a confidence level of 0.99 and *p* < 0.01.

The impact of chitosan on maintaining grain yield was evaluated in the two durum wheat genotypes and compared to the reference fungicides, and data are expressed as % of yield reduction relative to untreated control plants (without pre-treatment and *F. graminearum* inoculation) (Figure 7). Chitosan applied on Marco Aurelio’s spikes or flag leaves did not protect grain yield compared to *F. graminearum* inoculated plants. In fact, only tebuconazole applied directly on the spikes (62%) and the mixture of the reference fungicides applied on the spikes (59%) or on the flag leaves (65%) significantly contained the yield reduction compared to *F. graminearum* inoculated plants. On the other hand, chitosan applied on the flag leaves of DBC480 plants strongly contained the yield reduction (0.5%), as much as the azoxystrobin and the mixture of the reference fungicides applied on the spikes and the mixture of the reference fungicides applied on the flag leaf, compared to the *F. graminearum* inoculated DBC480 plants (20% of yield reduction).

**Figure 8:**
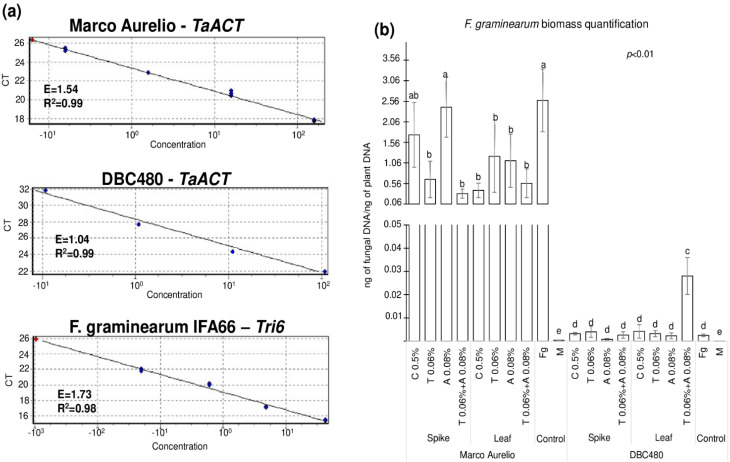
(**a**) Standard curves of the *TaACT* and *Tri6* genes for the Real-Time *q*PCR quantification of the fungal biomass. (**b**) *F. graminearum* biomass quantification expressed as ng of fungal DNA/ng of plant DNA from Marco Aurelio and DBC480 spikes recorded after treating the plants at the spike or flag leaf level with Chitosan at 0.5% (C 0.5%), tebuconazole at 0.06% (T 0.06%), azoxystrobin at 0.08% (A 0.08%), the mixture of tebuconazole at 0.06% and azoxystrobin at 0.08% (T 0.06% + A 0.08%) prior to *F. graminearum* inoculation, solely *F. graminearum* inoculated (Fg) and Mock (M) controls were included. The data represent averages and standard errors from four technical replicates derived from three biological replicates for each variable (wheat genotype × compound × mode of application). Different letters (a, b, c, d, e) into the subfigureb refer to the statistical analysis performed using three-way analysis of variance (ANOVA) with the Tukey test at a confidence level of 0.99 and *p* < 0.01.

Figure 8 shows the quantification of *F. graminearum* biomass measured by fungal DNA in the heads of Marco Aurelio and DBC480, where Figure 8a illustrates the three standard curves used for the quantification of durum wheat (*TaACT* gene) and *F. graminearum* (*Tri6* gene) biomass. E and R^2^ values ranged from 1.04 to 1.73 and from 0.98 to 0.99, respectively, from each primer pair. Figure 8b shows the results from the biomass quantification expressed as ng of fungal DNA/ng of plant DNA. Chitosan or azoxystrobin applied on Marco Aurelio’s spikes (1.74 and 2.41 ng of fungal DNA/ng of plant DNA, respectively) did not reduce the accumulation of the fungal biomass compared to the *F. graminearum* inoculated control (2.58 ng of fungal DNA/ng of plant DNA). On the other hand, the application of chitosan on the flag leaves in Marco Aurelio plants reduced the accumulation of the fungal biomass as much as the reference fungicides (0.39, 1.22, 1.11, and 0.56 ng of fungal DNA/ng of plant DNA from chitosan, tebuconazole, azoxystrobin, and the mixture of the reference fungicides treated heads). In DBC480 plants, neither the added compounds nor the mode of applications influenced the accumulation of the fungal biomass compared to the *F. graminearum* inoculated plants (0.0025 ng of fungal DNA/ng of plant DNA), with the exception of the mixture of the two reference fungicides applied at the flag leaf level, which surprisingly increased *F. graminearum* biomass into the heads (0.028 ng of fungal DNA/ng of plant DNA). In the mock-treated control heads, only traces of fungal DNA were detected (3.6 × 10^−4^ and 4 × 10^−5^ ng of fungal DNA/ng of plant DNA in Marco Aurelio and DBC480, respectively).

**Figure 9:**
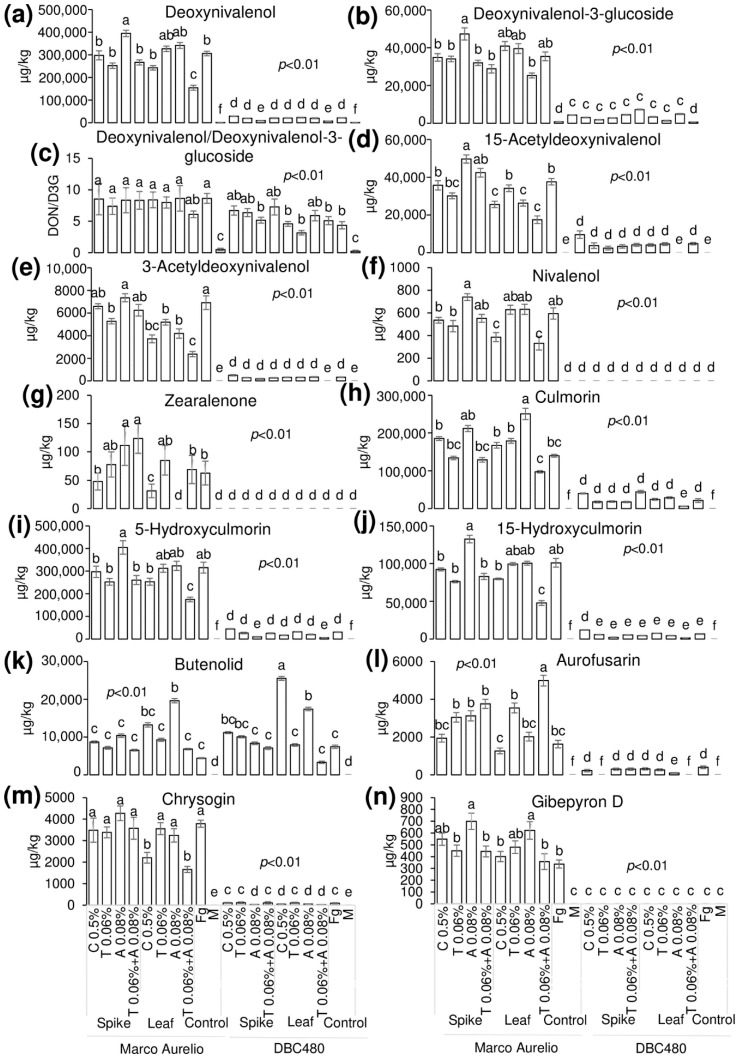
Detected and quantified mycotoxins and *F. graminearum*-associated molecules by using LC-MS/MS from Marco Aurelio and DBC480 spikes after treating the plants at the spike or flag leaf level with chitosan at 0.5% (C 0.5%), tebuconazole at 0.06% (T 0.06%), azoxystrobin at 0.08% (A 0.08%), the mixture of tebuconazole at 0.06% and azoxystrobin at 0.08% (T 0.06% + A 0.08%) prior to *F. graminearum* inoculation, solely *F. graminearum* inoculated (Fg) and Mock (M) controls were included as controls. (**a**) Deoxynivalenol; (**b**) Deoxynivalenol-3-glucoside; (**c**) Deoxynivalenol/Deoxynivalenol-3-glucoside; (**d**) 15-Acetyldeoxynivalenol; (**e**) 3-Acetyldeoxynivalenol; (**f**) Nivalenol; (**g**) Zearalenone; (**h**) Culmorin; (**i**) 5-Hydroxyculmorin; (**j**) 15-hydroxyculmorin; (**k**) Butenolid; (**l**) Aurofusarin; (**m**) Chrysogin; (**n**) Gibepyron D. The data represent averages and standard errors from four technical replicates derived from three biological replicates for each experimental group (wheat genotype × compound × mode of application). Different letters (a, b, c, d, e, f) into the subfigures refer to the statistical analysis performed using three-way analysis of variance (ANOVA) with the Tukey test at a confidence level of 0.99 and *p* < 0.01.

Liquid chromatography coupled to tandem mass spectrometry (LC-MS/MS) was applied to detect and quantify the FHB-associated mycotoxins and other FHB-related compounds in the durum wheat flours from the heads of Marco Aurelio and DBC480 subjected to the different compound treatments and modes of application (Figure 9). Deoxynivalenol (DON) (a) was not affected by the chitosan treatment in Marco Aurelio (297,000 µg/kg and 243,000 µg/kg by the spikes and flag leaves application, respectively), compared to the *F. graminearum* inoculated control (306,000 µg/kg). On the other hand, azoxystrobin applied on the spikes (395,000 µg/kg) favored its accumulation. In DBC480, chitosan did not influence the amount of detected DON (29,000 µg/kg and 20,700 µg/kg by the spikes flag leaves application, respectively) compared to the *F. graminearum* inoculated control (21,300 µg/kg). The amount of detected deoxynivalenol-3-glucoside (D3G) (b) was not affected by the compounds’ treatments and mode of applications in both Marco Aurelio and DBC480. The DON/D3G ratio (c) was calculated to evaluate the amount of DON transformed in D3G as a detoxification mechanism. The DON/D3G ratio was not influenced by the different compounds and mode of application in both the two durum wheat genotypes, but there was a significant difference between the two genotypes, since DBC480 showed lowest DON/D3G values compared to Marco Aurelio, after the following treatments: Azoxystrobin applied on the spikes (8.34 in Marco Aurelio and 5.17 in DBC480), chitosan applied on the flag leaves (8.41 in Marco Aurelio and 4.59 in DBC480), tebuconazole applied on the flag leaves (7.99 in Marco Aurelio and 3.17 in DBC480), and *F. graminearum* inoculated control plants (8.64 in Marco Aurelio and 4.35 in DBC480). The amount of 15-acetyldeoxynivalenol (15-ADON) (d) was reduced in Marco Aurelio after the application on the flag leaves of chitosan (25,600 µg/kg), azoxystrobin (26,300 µg/kg), and the mixture of the reference fungicides (17,500 µg/kg) compared to *F. graminearum* inoculated control (37,600 µg/kg). In DBC480, only the application of the reference fungicides on the flag leaves reduced the amount of 15-ADON, since the detected quantity was under the limit of detection (LOD) of the methodology, compared to *F. graminearum* inoculated control (4740 µg/kg). The detected amount of 3-acetyldeoxynivalenol (3-ADON) (e) was lower in Marco Aurelio in the flours derived from the application of chitosan, tebuconazole, azoxystrobin, and the mixture of the reference fungicides on the flag leaves (3720, 5190, 4190, and 2370 µg/kg, respectively) compared to the *F. graminearum* inoculated control heads (6910 µg/kg). On DBC480, only the mixture of the reference fungicides applied on the flag leaves decreased the amount of accumulated 3-ADON (under the LOD) compared to the *F. graminearum* inoculated control plants (326 µg/kg). Chitosan and the mixture of the reference fungicides applied on the flag leaves reduced the amount of nivalenol (NIV) (f) in Marco Aurelio (385 and 332 µg/kg, respectively) compared to the *F. graminearum* inoculated control (595 µg/kg), while NIV was not detected in all the DBC480 treated heads. Zearalenone (ZEA) (g) was significantly reduced in Marco Aurelio by the application on the flag leaves of chitosan (31 µg/kg), while its accumulation was favored by the application on the spikes of azoxystrobin (111 µg/kg) and the mixture of the reference fungicides (124 µg/kg) compared to the *F. graminearum* inoculated control (63 µg/kg). ZEA was not detected in all the DBC480 treated plants. The amount of detected culmorin (CUL) (h), of 5-hydroxyculmorin (5-HCUL) (i) and 15-hydroxyculmorin (15-HCUL) (j) was not reduced in Marco Aurelio and DBC480 after the application of chitosan compared to the *F. graminearum* inoculated plants. The application on the flag leaves of chitosan induced the accumulation of Butenolid (BUT) (k) in DBC480 heads (25,500 µg/kg) compared to the *F. graminearum* inoculated control (7430 µg/kg). The accumulation of aurofusarin (AUR) (l) in Marco Aurelio was reduced by the application of chitosan on the flag leaves (1260 µg/kg) compared to the rest of the applied treatments. On the other hand, chitosan did not reduce the amount of accumulated AUR in DBC480, compared to the *F. graminearum* inoculated control (398 µg/kg). Chrysogin (CHR) (m) was significantly reduced in Marco Aurelio after chitosan and the mixture of the reference fungicides applied on the flag leaves (2200 and 166 µg/kg, respectively), compared to the *F. graminearum* inoculated control (3790 µg/kg). On DBC480, azoxystrobin applied on the spikes (25 µg/kg) and chitosan (45 µg/kg), azoxystrobin (50 µg/kg), and the mixture of the reference fungicides (21 µg/kg) applied on the flag leaf contained the accumulation of CHR compared to the *F. graminearum* inoculated control (89 µg/kg). The amount of detected Gibepyron D (GID) (n) in Marco Aurelio was not influenced by the treatments with the exception of azoxystrobin applied on the spikes or on the flag leaves, which favored its accumulation (699 and 621 µg/kg, respectively) compared to the *F. graminearum* inoculated plants (336 µg/kg). In DBC480 GID was not detected (under the LOD).

**Figure 10:**
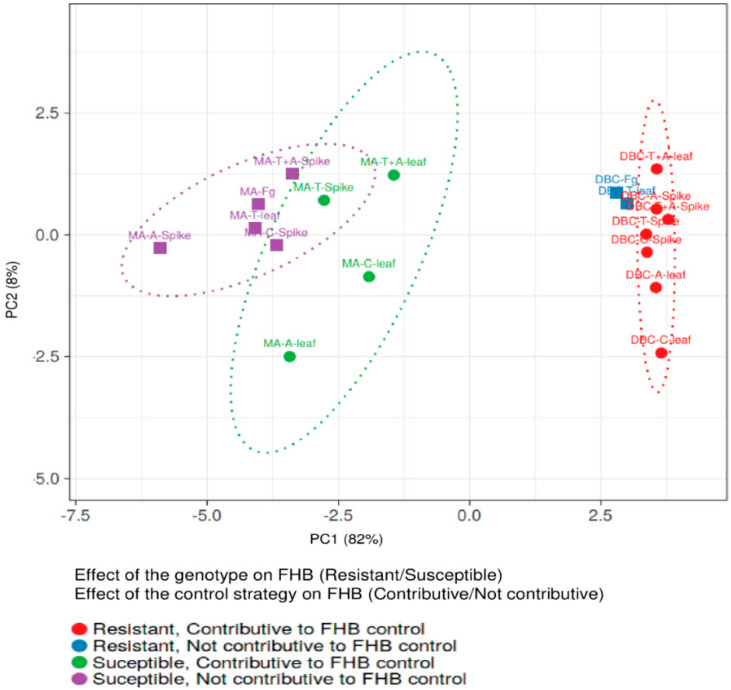
Principal Component Analysis (PCA) of the two durum wheat genotypes Marco Aurelio (MA) and DBC480 (DBC), plant treatments chitosan (C), tebuconazole (T), azoxystrobin (A), the mixture of tebuconazole and azoxystrobin (T + A), and the *F. graminearum* inoculated control (Fg), and the two modes of application (spike of leaf). The PCA was carried out to evaluate the genotype × compound effects on controlling FHB by plotting the % of FHB incidence and severity at 21 dpi, the % of yield reduction, the ng of fungal DNA/ng of plant DNA, and the µg/kg of the analyzed mycotoxins and *F. graminearum*-related compounds. PCA was conducted by using ClustVis software (Tartu, Estonia).

Notably, the % of yield reduction, the amount of fungal biomass, and FHB-related molecules were significantly lower in DBC480 than in Marco Aurelio, with the exception of DON/D3G ratio for chitosan, tebuconazole, and the mixture of the reference fungicides applied on the spike and azoxystrobin applied on the leaves, and the amount of detected BUT. In this regard, the interaction between the effect of the durum wheat genotypes and the control strategies in reducing FHB was evaluated by principal component analysis (PCA) (Figure 10). The two genotypes clearly clustered separately as susceptible (Marco Aurelio) and resistant (DBC480). Tebuconazole applied on the spikes and chitosan, azoxystrobin, and the mixture of the reference compounds applied on the flag leaves, contributed to FHB control in Marco Aurelio, while all the treatments, with the exception of tebuconazole applied on the flag leaves, contributed to FHB control in DBC480.

## 3. Discussion

The concerns regarding the environmental safety and the efficacy of fungicides in controlling FHB highlight the urgent need to develop green as much as possible and environmentally safe fungicides that are effective in reducing FHB and mycotoxins accumulation in kernels with different modes of action from that of conventional molecules [17]. In this regard, chitosan was extensively evaluated as a plant biostimulant and antimicrobial agents against different plant pathogens (fungi, bacteria, and viruses), and recently against different *Fusarium* spp. [51,52,53,54], and the modes of action have been hypothesized to describe its ability to boost plant growth and defense mechanism [39,50]. Many studies evaluated the antifungal ability of chitosan against FHB pathogens in vitro and in vivo. A recent study [51] evaluated in vitro and in vivo chitosan oligomers mixed with amino acids against *F. culmorum,* reporting that chitosan oligomers demonstrated a 90% maximal effective concentration (EC_90_) of 2230.26 µg/mL (0.2%). These results are in agreement with those obtained in the present study because we also observed an inhibition very close to 90% at a similar chitosan concentration (0.1%). Interestingly, the authors obtained valuable data on the reduction of disease severity and DON content by mixing chitosan with tyrosine. Kheiri et al. (2016, 2017) tested chitosan and chitosan nanoparticles on *F. graminearum* growth and they observed the highest inhibition in vitro by supplementing PDA with chitosan at 0.1% (68% of growth inhibition) and with chitosan nanoparticles at 0.5% (85% of growth inhibition), compared to the reference tilt fungicides applied at the recommended field dose (72% of growth inhibition). Interestingly, the microscopic examination of the treated *F. graminearum* showed dehydration and deformation in mycelial growth. During the greenhouse experiments, by using as host plant the cultivar Falat (bread wheat) they observed a disease severity of 29% and 27% in chitosan and chitosan nanoparticles treated plants, while the plants treated with the tilt fungicides reached 0.33% of disease severity after three weeks of inoculation [55,56]. Another study [57] combined chitosan with a plant biostimulant, the liquid seaweed extract (LSE) from a brown macroalga and evaluated the efficacy of chitosan and LSE, alone or combined, against *F. graminearum* growth. In vitro, no synergistic effect of LSE and chitosan was observed on mycelial growth and chitosan at 0.001% showed 43% of growth inhibition. On the other hand, during the in vivo experiments (cultivar Helios, bread wheat), the authors observed a synergistic effect of LSE and chitosan in reducing FHB severity, while chitosan applied alone reduced FHB severity of 15% compared to the inoculated control plants. Notably, the authors also evaluated the induction of transcription of *TaPR1*, *TaPR2,* and *TaPR3* in wheat seedlings at 24, 48, and 72 h post inoculation (hpi). *TaPR1* and *TaPR2* showed the greatest up-regulation at 48 hpi (25 and 500-fold, respectively) and were significantly induced in all the chitosan-based treatment compared to the plants subjected only to *F. graminearum* inoculation (10 and 200-fold, respectively) [57]. These results are in agreement with data obtained from our experiments since we observed a consistent *F. graminearum* growth inhibition by applying chitosan in vitro at different concentrations (0.01–1%) and a significant reduction of FHB incidence and severity during the greenhouse experiments by applying chitosan at 0.5%. Moreover, we also observed a significant up-regulation of defense-related genes at 48 hpi (*TaPAL*, *TaPR1,* and *TaPR2*) in chitosan treated plants and *F. graminearum* inoculated spikes, even though we observed a transcript amount extremely low if compared to the fold-change detected by the previously cited authors. These differences could be attributed to the fact that the authors used as host plant a bread wheat cultivar while we performed the experiments by using two durum wheat genotypes; thus, the abundance of the transcript amounts assigned to the D genome of bread wheat are absent in the durum wheat genotypes. Furthermore, they evaluated the relative expression of the genes from treated seedlings, while we analyzed the transcript on the spikes, which were not subjected directly to the treatments, since the application of the compounds was performed on the flag leaves to observe the putative indirect antifungal ability of chitosan as SAR inductor. Regarding the antimicrobial mechanism of chitosan, several researchers have presented their hypotheses on the direct and/or indirect effects against pathogens: The positive charge of the protonated chitosan enables electrostatic interaction with the negative charge of the pathogens surface; chitosan increases the permeability of the membrane by damaging the cells of the pathogens resulting in cell death [58]; chitosan is also able to chelate the essential elements for the growth of the pathogens, such as metal ions, minerals, and nutrients, preventing the normal growth of the pathogens; chitosan may also interact with DNA or RNA leading to the inhibition of the mRNA synthesis; the deposition of chitosan on plant and pathogen surface forms a biofilm that limits the nutrient availability for microorganisms [59,60,61]; the ability of chitosan to induce the SAR in plants is associated with its elicitor-like properties, which boost the early activation of pathogenesis-related proteins and improve the plant resistance. As a result, chitinase and β-1,3-glucanase (PR2) enzymes degrade the fungal cell wall and stop its growth on the host plant [39,50,61].

During the in vivo evaluation of the antifungal activity, a significant reduction of FHB incidence and severity was observed in Marco Aurelio and DBC480 plants treated at the flag leaves with azoxystrobin-based formulations. The ability of azoxystrobin applied on the flag leaves to protect the plants against FHB was also confirmed by the basal-regulation in Marco Aurelio of *TaPAL* and *TaPR1*, the up-regulation of *TaPAL* and *TaPR1,* and the basal-regulation of *TaPR2* in DBC480 and by the reduced amount of some types of mycotoxins accumulated in the flours. *TaPAL* is the key enzyme in the biosynthesis of salicylic acid (SA), which is the central player in the hypersensitive reaction (HR) and SAR, which are associated with early signaling events in response to pathogens, such as the activation of PR genes [62,63,64,65]. Many studies demonstrated that the fast activation of SAR by expressing diverse PR genes resulted in a significant enhancement of FHB resistance [66,67,68]. Moreover, it was demonstrated that strobilurins improve the hormonal balance in plants. In particular, z high concentration of strobilurins inhibits the biosynthesis of ethylene (ET) [69] but improves the production of abscisic acid (ABA) [70]. It has been demonstrated that ET contributes to FHB resistance during early infection but then promotes the senescence of wheat heads that favors the fungal colonization in the dead tissues of the host [71,72]. On the other hand, the accumulation of ABA quickly induces the stomatal closure, which prevents the pathogen entry into the host tissues [73,74]. For such these reasons, the concomitant ability of azoxystrobin to induce the SAR pathway, to inhibit the ET biosynthesis, and favor ABA accumulation could have led to the reduction of FHB incidence and severity as much as the mycotoxin’s accumulation in Marco Aurelio and DBC480 plants.

Chitosan was tested as a plant growth-promoter in many crops in several research studies. Indeed, the chitosan seeds coating resulted to boost the germination rate, the root apparatus, the aerial biomass, the photosynthetic rate, and the yield in maize, pearl millet, rapeseed, soybean, sunflower, peanut, rice, tomato, chickpea, and coffee [75,76,77,78]. In wheat, the kernels coated with chitosan (0.2–0.8%) significantly improved the germination rate, seedling growth parameters, and the synthesis of antioxidant enzymes (superoxide dismutase, peroxidase, and catalase) [79]. Ma et al. (2012) reported that wheat kernels treated with chitosan showed higher growth than control under salinity stress [80]. These results are in agreement with the data presented in the present study, since chitosan positively influenced the growth parameters on Marco Aurelio and DBC480 after coating the kernels with chitosan at 0.5%. The chitosan’s ability to boost the germination rate in many plant species has been attributed to its excellent film forming property, allowing it to form a semipermeable film on the seed surface, which can maintain the seed moisture and absorb the soil moisture, promoting the seed germination [79]. Furthermore, the signaling mechanism of chitosan to regulate the development and growth in plants has been elucidated on various crops [75,76,77,78]. Chitosan helps in the activation of hydrolytic enzymes required for the degradation of reserve nutrients such as starch [78]. Chitosan stimulates root cell division by the activation of auxin and cytokinin, which further contribute to the uptake of nutrients. Chitosan also significantly enhanced the crop yield by boosting the photosynthesis index by increasing the chlorophyll content and stomatal activity by switching on the genes related to the chlorophyll synthesis [78].

In the present study, we demonstrated that chitosan inhibited, in vitro, the *F. graminearum* growth, virulence, and trichothecenes synthesis by analyzing the transcripts amount of the major genes involved in cell growth, respiration, establishment of virulence, and mycotoxins biosynthesis. To our knowledge, this is the first research study evaluating the *F. graminearum* genes response after treating the mycelium with chitosan and reference fungicides (tebuconazole and azoxystrobin). We also demonstrated that chitosan at 0.5% contained the fungal spread inside the head as much as the accumulation of various mycotoxins and *F. graminearum*-related compounds, especially if applied at the flag leaf level, in the two durum wheat genotypes (Marco Aurelio and DBC480). No reports regarding the quantification of the FHB-associated pathogens in the spikes after chitosan treatment have been performed before, thus this is the first study that assesses the ability of chitosan to control the *F. graminearum* spread in the heads applied directly on the spikes or on the flag leaf. On the other hand, two studies evaluated the efficacy of tebuconazole and azoxystrobin in controlling FHB-associated pathogens spread by preventively applying the compounds on the spikes. Both the research studies demonstrated that tebuconazole significantly reduced the amount of fungal DNA in the spikes, while azoxystrobin was completely ineffective [81,82]. These results are in agreement with those obtained in the present study, were tebuconazole and the mixture of tebuconazole and azoxystrobin applied on the spikes of Marco Aurelio reduced the accumulated fungal biomass, while the amount of fungal DNA detected from azoxystrobin treated heads were similar to the *F. graminearum* inoculated control.

Khan et al. (2009) and Zachetti et al. (2018) quantified the amount of DON from inoculated kernels after treating the spikes with chitosan, which led to a significant reduction of the mycotoxin [83,84]. Another study quantified DON, D3G, 15-ADON in spikes subjected to different chitosan-based treatments and they also observed a reduction in the number of accumulated mycotoxins [57]. These results agree with those obtained in the present study, since chitosan, especially when applied at the flag leaf level, reduced the amount of most of the mycotoxins analyzed in the present work. Many studies also evaluated the effect of tebuconazole and azoxystrobin in the reduction of DON content, highlighting that only tebuconazole is effective in controlling the accumulation of DON in the spikes, while azoxystrobin resulted ineffective or favored the accumulation of a higher quantity of DON [21,22,82,85,86]. The present research study confirms the results previously obtained, since tebuconazole contributed to decrease the number of accumulated mycotoxins while azoxystrobin applied on the spikes did not influence it or in many cases favored their accumulation. To our knowledge, no reports regarding the accumulation of 3-ADON, NIV, ZEA, CUL, 5-HCUL, 15-HCUL, BUT, AUR, CHR, and GID in response to chitosan or reference fungicides is present. Thus, this is the first research work that analyzed the amount of these FHB-related compounds after chitosan, tebuconazole, and azoxystrobin treatments.

During all the experiments, there was a significant difference between Marco Aurelio and DBC480 in the ability to counteract FHB. *F. graminearum* inoculated control in DBC480 plants demonstrated a strong type II resistance (resistance to infection spread) to FHB, since FHB severity was of 25% at 21 dpi, the yield reduction was around 20%, and DBC480 inoculated plants accumulated a significantly lower amount of *F. graminearum* biomass and mycotoxins compared to Marco Aurelio. The resistant behavior of DBC480 is partly attributed to the presence of *Fhb1*. *Fhb1* is one of the most effective QTL in contrasting FHB and derives from the Chinese Spring wheat of Sumai3. *Fhb1* is located on the short arm of the chromosome 3B and provides type II resistance. Nowadays, the identity and mode of action of *Fhb1* are not fully clarified, in fact, many research studies attributed the identity of *Fhb1* to diverse genes, but many evidences underlined that *Fhb1* is involved in DON detoxification [87,88,89]. This hypothesis is also supported by the present study on the evidence that the DON/D3G ratio was lower in DBC480 compared to Marco Aurelio. Interestingly, the application of chitosan on DBC480 plants decreased FHB severity to 6% at 21 dpi compared to 25% of FHB severity of the *F. graminearum* inoculated control plants, suggesting that the strategy of combining a resistant genotype with a novel and sustainable active ingredient shows additive effects and appears effective for the concomitant reduction of FHB and xenobiotic compounds in the environment.

## 4. Materials and Methods

### 4.1. Fungal, Chemical and Plant Materials

The highly virulent and mycotoxin-producing isolate of *F. graminearum* IFA66 was originally isolated by the IFA-Tulln (Tulln an der Donau, Austria) [90], and was the reference strain used in this study. Chitosan hydrochloride (99% purity, molecular weight 60 kDa, degree of deacetylation 80–90%) (CAS number 70694-72-3), a water-soluble derivate of chitosan, tebuconazole (CAS number 107534-96-3), and azoxystrobin (CAS number 131860-33-8) were purchased from Merck (Darmstadt, Germany). The FHB-susceptible Italian durum wheat cultivar Marco Aurelio was provided by the Agricultural Consortium of Perugia (Umbria Region, Italy). This genotype, grown in Central and South of Italy, is extensively used for pasta production and it is characterized by an excellent protein content and high productivity, thus it has a high economic importance. The FHB-resistant durum wheat experimental line DBC480, harboring the resistant quantitative trait locus *Fhb1*, was provided by IFA-Tulln (Tulln an der Donau, Austria). DBC480 was developed at IFA-Tulln by four generations of marker-assisted backcrossing of the highly resistant *T. aestivum* cultivar Sumai3 into the background of the Austrian *T. durum* variety Semperdur [91].

### 4.2. In Vitro Assays

Four different in vitro assays were performed in order to test the inhibitory effect of chitosan hydrochloride against *F. graminearum.* Five concentrations of chitosan hydrochloride have been assayed, 0.01–0.05–0.1–0.5, and 1% (*w*/*v*). Moreover, tebuconazole and azoxystrobin were tested alone or in combination as reference fungicides at the suggested field dose from commercial formulations (0.06% *w*/*v* for tebuconazole and 0.08% *w*/*v* for azoxystrobin).

#### 4.2.1. 96 Microtiter Plates Assay and Determination of the Minimum Inhibitory Concentration (MIC)

Fresh mycelium of *F. graminearum* grown on Potato Dextrose Agar (PDA) was transferred on Synthetic Nutrient Poor Agar (SNA) and cultured at 21 °C to obtain macroconidia [92]. After 10 days on SNA, the conidia were scraped with a glass rod after pipetting 1 mL of sterile distilled water onto the surface of a Petri dish. The conidial suspension was recovered, and the concentration was measured by using a Thoma Chamber (0.100 mm depth and 0.0025 mm^2^). Chitosan hydrochloride, tebuconazole, and azoxystrobin were dissolved in sterile distilled water at the previously cited concentrations, the pH values were controlled and resulted to be around 5, and filtered by using a sterile syringe filter with a pore size of 0.2 µm. The obtained solutions were pipetted into the microtiter plates and the conidial suspension was added to each well in order to obtain a final concentration of 1 × 10^5^ conidia/mL. The plates were incubated at 21 °C in the dark for 24 h. After that, 10 µL of the conidial-antimicrobial compound suspension were transferred into a new microtiter plate containing Potato Dextrose Broth (PDB) and incubated at 21 °C in the dark for 48 h. The absorbance from the fungal biomass was measured at OD_450_ by using a DR-200B Microplate reader (Diatek instruments). Mock (untreated conidial suspension cultured on PDB) and blank (PDB) controls were also included. The % of growth inhibition was calculated for each tested substance and concentration by using the following equation [93,94]:

% of growth inhibition = 100 × (Mock-Treated/Mock).(1)

Data were obtained from three independent experiments, each one consisting of four replicates for each substance and concentration tested.

#### 4.2.2. Incorporated Medium Assay

Sterile PDA was supplemented with chitosan hydrochloride, tebuconazole, azoxystrobin, or a mixture of tebuconazole and azoxystrobin at the previously cited concentrations. The PDA incorporated with the compounds was poured on Petri dishes and allowed to solidify. Afterward, a 5 mm plug from a fresh culture of *F. graminearum* was placed at the center of the Petri dish and cultured in the dark at 21 °C for 7 days. Mock control was also included. After 7 days, the mycelial growth was evaluated by measuring four fungal colony diameters from each plate. The % of growth inhibition was calculated for each tested substance and concentration by using the Equation (1) [94].

Data were obtained from three independent experiments, each one consisting of four replicates for each substance and concentration tested.

#### 4.2.3. Agar Diffusion Assay

A *F. graminearum* conidial concentration of 1 × 10^5^ conidia/mL was obtained and prepared as described in the “96 microtiter plates assay and determination of MIC” paragraph. One milliliter of the conidial suspension was homogenously plated on the surface of PDA onto the Petri dishes and allow to dry. After that, a well of 5 mm diameter was created by punching the agar in the center of the Petri dish and 100 µL of the tested compounds at different concentrations were pipetted inside. Mock control was also included. The plates were incubated in the dark at 21 °C for 48 h. Afterward, the visible inhibition halo was evaluated by measuring four diameters from each plate. The % of growth inhibition was calculated for each tested substance and concentration by using the following equation [94]:

% of growth inhibition = 100 × (Treated-Mock/Mock).(2)

Data were obtained from three independent experiments, each one consisting of four replicates for each substance and concentration tested.

#### 4.2.4. Real-Time qPCR

The relative expressions of the genes regulating the cellular physiology, the virulence, and the trichothecenes production in *F. graminearum* were evaluated after treating the mycelium with chitosan hydrochloride, tebuconazole, azoxystrobin, or the mixture of tebuconazole and azoxystrobin. First, solutions of PDB supplemented with chitosan hydrochloride at 1% *w*/*v*, tebuconazole at 0.06% *w*/*v*, azoxystrobin at 0.08% *w*/*v,* or the mixture of tebuconazole at 0.06% *w*/*v* and azoxystrobin at 0.08% *w*/*v* were prepared in sterile glass flasks. Starting from these solutions, four serial dilutions (1:10, 1:100, 1:1000, 1:10,000) were prepared for each compound. The dilutions were performed in PDB, in order to identify the optimal concentration that allowed to obtain the same quantity of fungal biomass compared to the mock treatment. In this way, the detected transcripts amount was not influenced by the recorded fungal biomass. One milliliter of a conidial suspension at 1 × 10^5^ conidia/mL of *F. graminearum* was pipetted in each flask and cultured in the dark at 21 °C in an orbital shaker for 48 h. After that, 1 mL of each suspension was measured spectrophotometrically (OD_450_) (Onda Spectrophotometer), and the serial dilution (1:1000) allowed to reach the same absorbance value of the mock treatment was chosen for the transcript’s analysis. Fresh PDB was used as blank control. The fungal biomass was recovered by using a vacuum pump from the 1:1000 dilutions for each antimicrobial compound and immediately stored in liquid nitrogen and at −80 °C. One hundred milligrams of mycelium were subjected to RNA extraction following the instructions provided by InviTrap^®^ Spin Plant RNA Mini Kit (Stratec Molecular). The RNA was resuspended in RNase-free sterile distilled water and immediately poured onto ice and quantified with Qubit™ fluorometer 1.01 (Invitrogen, Carlsbad, CA, USA) using the Qubit™ RNA BR Assay Kit (Thermo Fisher Scientific, Waltham, MA, USA). To confirm the total quantity and integrity of the RNA, 5 µL of the extracted RNA was subjected to thermal shock (10 min at −80 °C after 5 min at 65 °C) and run on 1.5% denaturating agarose gel. The synthesis of the cDNA was performed using 500 ng of RNA following the instructions provided by Xpert cDNA Synthesis Supermix with a gDNA eraser (GRiSP Research Solutions) in a final volume of 20 µL. To ensure that the synthesis of the cDNA and the elimination of the gDNA had succeeded, a reverse transcriptase PCR (RT-PCR) of the translational elongation factor 1-α (*TEF*) (containing an intron in the amplified sequence) was performed. The RT-PCR was performed by following the instructions provided by GoTaq MasterMix (Promega) in a total volume of 10 µL and conditions of amplification included: An initial denaturation step of 2 min at 95 °C; 35 cycles of 30 s denaturation at 95 °C; 40 s of annealing at 53 °C; 30 s of elongation at 72 °C; a final elongation step of 5 min at 72 °C. The amplification run also consisted of a no-template control (NTC) and a genomic DNA (gDNA) control. The amplicons were visualized on 1.5% agarose gel. Table S3 shows the list of target genes, accession numbers, their functions, the corresponding primer pairs, and their references used to perform the RT-*q*PCR [95,96,97,98,99,100,101,102]. The efficiency of the RT-*q*PCR amplification (E) was determined for each primer pair as follows: Five 1:10 serial dilutions (1:1-1:10,000) were obtained for each cDNA and amplified in four replicates. E and correlation coefficient (R^2^) values were calculated by means of the slope of the standard curve obtained by plotting the fluorescence versus the serial dilution concentrations using the equation:

E = 10^(−1/slope)^ − 1 (3)

The relative expression levels of the target genes were calculated on the basis of the Cq values of the four technical replicates derived from four independent biological replicates for each compound by applying the equation:

Relative expression = 2^−ΔΔCq^ (4)

using *TEF, TUB,* and *GAPDH* as the reference genes and the mock treatment to normalize the relative expression levels [103]. The RT-*q*PCR was performed following the instructions provided by the Rotor Gene Q (Qiagen, Hilden, Germany and Xpert Fast SYBR (uni) MasterMix (Grisp, Porto, Portugal), in a final volume of 10 µL. The amplification conditions included an initial denaturation step of 3 min at 95 °C; 40 cycles of 5 s denaturation at 95 °C; 30 s of annealing at 60 °C; and 20 s of elongation at 72 °C. A final melt cycle (70–99 °C) was performed to confirm the amplicons’ unicity. NTC controls were included and the amplification was considered negative when a value of Cq ≥ 38 was detected.

### 4.3. Coating of Kernels and Evaluation of the Biostimulant Effect

The evaluation of the biostimulant effect of chitosan hydrochloride was conducted in a glass house located in Viterbo (Experimental Farm of the University of Tuscia, Viterbo, Italy), in order to verify if chitosan was able to boost the plant grow. For this assay, chitosan hydrochloride at 0.5% was tested, since this was the most promising concentration from the previous in vitro experiments. The solution was prepared in sterile distilled water as previously described. The surface of the kernels of the durum wheat genotypes Marco Aurelio and DBC480 were sterilized with sodium hypochlorite (0.5% *v*/*v*) for 20 min and then rinsed twice for 5 min in sterile distilled water. After that, the kernels were soaked for 1 h in the solution containing chitosan and in distilled water as mock control under constant stirring and allowed to dry for 1 h. Then, the kernels were transferred to 40 × 20 cm pots, filled with TYPical Brill soil, and grown at 18/12 °C (day/night) with 12–14 h of light for 14 days. At the end of the experiment, the germinated kernels were counted, and a kernel was considered as germinated when the coleoptile was visible. Nitrogen Balance Index (NBI) (DUALEX Scientific+™) related to the chlorophyll and flavonoid ratio was measured by positioning the instrument in the center of the leaf and the meter was shielded from direct sunlight. Afterward, the seedlings were removed from the soil and gently washed with distilled water and the following growth parameters were measured: Coleoptile length (mm), main root length (mm), and number of developed roots. The % of growth promotion was calculated for each growth parameter measured (kernel germination, NBI, coleoptile and main root length, and number of developed roots) and tested substance by using the following equation [104]:

% of growth promotion = 100 × (Treated-Mock/Mock).(5)

Data were obtained from three independent experiments organized as a complete randomized block, each one consisting of 20 plants for each experimental group (wheat genotype × compound).

### 4.4. In Vivo Antifungal Activity Evaluation

The artificial inoculation trials were conducted in a glass house located of the University of Natural Resources and Life Sciences, Vienna, Department of Agrobiotechnology Tulln, Austria. Seeds of the durum wheat genotypes Marco Aurelio and DBC480 were germinated into pots (17 cm diameter, 20 cm height) containing a mixture of peat, compost, and sand and placed in the greenhouse. Temperature in the greenhouse was on average 18/12 °C (day/night) from tillering to heading with 12–14 h of light. During flowering time, the conditions in the greenhouse were controlled and set at 21 °C, 55% relative humidity during daytime and 17 °C, 55% humidity during night with a 16 h photoperiod. Chitosan hydrochloride at 0.5%, tebuconazole at 0.06%, azoxystrobin at 0.08%, and the mixture of tebuconazole 0.06% and azoxystrobin 0.08% were prepared in distilled water as previously described. Macroconidia of the single-spore *F. graminearum* IFA66 were produced in liquid mungbean medium as described by Buerstmayr et al. (2000, 2002) [105,106]. The mungbean medium was removed from the conidial suspension by multiple centrifugation steps in double-distilled water. Aliquots of conidia stock solutions were stored at −80 °C then thawed at 37 °C and diluted with distilled water to achieve the desired final spore concentration just prior to inoculation. The antifungal compounds were sprayed on plants (wetted until runoff) 48 h before inoculation in two different ways: On the spikes, to test the effective antifungal efficacy of the compounds, or on the flag leaves, to test the ability of boosting the SAR on durum plants. Plants subjected only to artificial inoculation and mock treatment were sprayed with distilled water. Plants were subjected to artificial inoculation at anthesis (Zadok stage 69) [107] by homogenously spraying a conidial suspension of 25,000 conidia/mL, while mock plants were sprayed with distilled water (wetted until runoff). The wheat heads were covered with plastic bags for 48 h to maintain high humidity levels (>80%). The disease incidence and severity of the FHB (%) were determined by counting the number of diseased spikes and the number of bleached spikelets in diseased spikes as well as the total number of spikes and spikelets, respectively, from 3 to 21 dpi. Data were obtained from three independent experiments organized as a complete randomized block, each one consisting of 10 plants for each experimental group (wheat genotype × compound × mode of application).

### 4.5. Evaluation of Induction of SAR by Real-Time qPCR

The relative expressions of the *TaPR1*, *TaPR2* (pathogenesis related protein 1 and 2), and *TaPAL* (phenylalanine ammonia lyase) were evaluated as involved in the biosynthesis of salicylic acid (*TaPAL*) and indicators of the induction of SAR (*TaPR1* and *TaPR2*). The flag leaves were pre-treated 48 h before the inoculation. The spikes were harvested 48 h after the inoculation and immediately stored in liquid nitrogen and at −80 °C. One hundred milligrams of fine powder were subjected to RNA extraction following the instructions provided by the RNeasy Plant Mini Kit (Qiagen). The RNA was resuspended in RNase-free sterile distilled water and immediately poured onto ice and quantified with NanoDrop™. To confirm the total quantity and integrity of the RNA, 5 µL of the extracted RNA was subjected to thermal shock (10 min at −80 °C after 5 min at 65 °C) and run on 1.5% denaturating agarose gel. The synthesis of the cDNA was performed using 5 µg of RNA following the instructions provided by the RevertAid First Strand cDNA Synthesis Kit (Thermo Scientific) in a final volume of 20 µL. To ensure that the synthesis of the cDNA and the elimination of the gDNA had succeeded, a reverse transcriptase PCR (RT-PCR) of the *TaACT* (containing an intron in the amplified sequence) was performed. The RT-PCR was performed as previously described in the “Real-Time qPCR” paragraph of the in vitro assays section by using an annealing temperature of 60 °C. The amplification run consisted also of a no-template control (NTC) and a genomic DNA (gDNA) control. The amplicons were visualized on 1.5% agarose gel. Table S4 shows the list of target genes, accession numbers, their functions, the corresponding primer pairs, and their references used to perform the RT-*q*PCR [108,109,110,111]. The efficiency (E) and correlation coefficient (R^2^) of the RT-*q*PCR were determined as previously described. The relative expression levels of the target genes were calculated on the basis of the Cq values of the four technical replicates derived from three independent biological replicates for each wheat genotype and compound by applying the Equation (4), using *TaFNR, TaTUB,* and *TaACT* as the reference genes and the mock treatment to normalize the relative expression levels [103]. The RT-*q*PCR was performed following the instructions provided by the CFX384 Touch Real-Time PCR Detection System (Bio-Rad) and iTaq™ Universal SYBR^®^ Green Supermix (Bio-Rad) in a final volume of 10 µL. The amplification conditions included an initial denaturation step of 30 s at 95 °C; 40 cycles of 10 s denaturation at 95 °C; 35 s of annealing and elongation at 60 °C. A final melt cycle (70–99 °C) was performed to confirm the amplicons’ unicity. NTC controls were included and the amplification was considered negative when a value of Cq ≥ 38 was detected.

### 4.6. Quantification of Fungal Biomass, Mycotoxins Analysis, and Impact on Yield

At the end of ripening, all the inoculated and mock spikes were collected in order to evaluate if the different compounds and modes of application (on the spike or on the flag lead) influenced the amount of fungal biomass and mycotoxins inside the flour and the impact on grain yield in the two durum wheat genotypes.

#### 4.6.1. Determination of the Impact on Grain Yield

Thirty spikes from each experimental group (wheat genotype × compound × mode of application) (10 spikes from each independent replicate) were harvested and weighed (g). The % of yield reduction was calculated by using the following equation:

% of yield reduction = 100 × (Mock-Treated/Mock)(6)

#### 4.6.2. Quantification of Fungal Biomass by Real-Time qPCR

Ten spikes from each independent replicate of each experimental group (wheat genotype × compound × mode of application) were homogenously milled into a fine flour. The isolate of *F. graminearum* IFA66 was re-isolated by plating 100 mg of the flour obtained from the inoculated and untreated spikes on PDA and cultured in the dark at 21 °C for 7 days in purity. One hundred milligrams of fresh mycelium and 100 mg of flour from Marco Aurelio and DBC480 samples were subjected to total DNA extraction following the protocol for the Invisorb^®^ Spin Plant Mini Kit (Stratec Molecular). DNA was quantified with a Qubit™ fluorometer 1.01 (Invitrogen) using the Qubit™ dsDNA BR Assay Kit (Thermo Fisher Scientific). DNA from inoculated samples was diluted to 10 ng/µL, while fungal and wheat calibration curves were obtained preparing four serial 1:10 dilutions (1:1, 1:10, 1:100, 1:1000) from fresh fungal mycelium and uninoculated wheat material DNAs. RT-*q*PCR was performed following the instructions from Rotor Gene Q (Qiagen) and Xpert Fast SYBR (uni) Master Mix (Grisp). RT-*q*PCR amplification conditions included: An initial denaturation step of 3 min at 95 °C; 40 cycles of 5 sec denaturation at 95 °C, 30 sec of annealing at 60 °C; and 20 sec of elongation at 72 °C. A final melt cycle (70–99 °C) was performed to confirm the amplicons unicity. RT-*q*PCR was performed using the primer pair Tri6_10F/Tri6_4R for *F. graminearum* DNA quantification and TaACT_F/TaACT_R for wheat DNA quantification (Tables S3 and S4). Data were obtained from four technical replicates derived from three biological replicates. Results were expressed as ng of fungal DNA per ng of plant DNA [98,112].

#### 4.6.3. Detection and Quantification of Mycotoxins by Liquid Chromatography Coupled to Tandem Mass Spectrometry (LC-MS/MS)

Five grams of homogenized sample were extracted with 40 mL of acetonitrile/water/acetic acid (79:20:1, *v/v/v*) solvent in a 50 mL tube for 90 min and 180 rpm shaking speed using a GFL 3017 rotary shaker (GFL 2017). The raw extract was diluted 1:20 using acetonitrile/water/acetic acid (20:79:1, *v/v/v*) and 5 µL of diluted extract were injected subsequently. Metabolite analysis was carried out using a 1290 Series HPLC System (Agilent Technologies, Santa Clara, CA, USA coupled to a QTrap 5500 LC-MS/MS System (Applied Biosystems SCIEX, Foster City, CA, USA) equipped with Turbo Ion Spray electrospray ionization source. Chromatographic separation was performed at 25 °C on a Gemini^®^ C_18_-column, 150 × 4.6 mm i.d., 5 µm particle size, equipped with a C_18_ 4 × 3 mm i.d. security guard cartridge (Phenomenex, Torrance, CA, USA). Confirmation of positive metabolite identification was carried out by the acquisition of two-time scheduled multiple reaction monitoring (MRMs), which yielded 4.0 identification points according to the European Commission decision 2002/657. In addition, retention time and ion ratio had to agree with the related values of authentic standard within 0.03 min and 30% rel., respectively. Results were corrected using apparent recoveries obtained during validation. The accuracy of the method is verified on a continuous basis by participation in a proficiency testing scheme organized by BIPEA (Gennevilliers, France) with a current rate of z-scores between −2 and 2 of >94% (>1300 results submitted) [113].

### 4.7. Statistical Analysis

Data from in vitro experiments, RT-*q*PCR of the *F. graminearum* genes, biostimulant evaluation, and FHB disease incidence and severity were subjected to one-way analysis of variance (ANOVA), while data from RT-*q*PCR of the durum wheat genes were subjected to two-way ANOVA where the two independent variables were the wheat genotypes (Marco Aurelio and DBC480) and the treatments applied to the plants (chitosan 0.5%, tebuconazole 0.06%, azoxystrobin 0.08%, tebuconazole 0.06% + azoxystrobin 0.08%, and *F. graminearum* inoculated plants) and the dependent variable was the relative expression level. Data from yield reduction, fungal biomass, and mycotoxins quantification were subjected to three-way ANOVA where the three independent variables were the wheat genotypes, the plant treatments, and the mode of application of the compounds on plants (application on the spike or on the flag leaf). One level of significance (*p* < 0.01) was computed to assess the significance of the F values. A pairwise analysis was carried out using the Tukey Honestly Significant Difference test (Tukey test) at the 0.99 confidence level. Statistical analyses were performed using SYSTAT12 software (Systat Software Inc., San Jose, CA, USA). The heatmaps were generated to represent the relative expression levels of *F. graminearum* and wheat genes after the plant treatments, while the principal component analysis (PCA) was carried out to evaluate the genotype × compound effects on controlling FHB by plotting the % FHB incidence and severity at 21 dpi, the % of yield reduction, the ng of fungal DNA/ng of plant DNA, and the µg/kg of the analyzed mycotoxins and *F. graminearum*-related compounds. PCA and heatmap were carried out using ClustVis software (Tartu, Estonia) [114].

## 5. Conclusions

Chemical fungicides accumulate in the soil, being part of the animal and human food chain, causing negative or unknown effects on the health. Furthermore, the efficiency of tebuconazole and azoxystrobin in protecting wheat plants from FHB-related pathogens is extremely variable and their constant use is favoring the selection of resistant strains. Chitosan is a natural, biodegradable, non-toxic, and eco-friendly compound with a wide range of positive properties for plants, such as biostimulator of growth and of defense systems. The present study demonstrated that chitosan hydrochloride, a water-soluble form of chitosan, boosted the wheat growth and development, reduced FHB severity, and the accumulation of the fungal biomass as much as the amount of several mycotoxins and FHB-related compounds in the spikes. The application of chitosan on the flag leaves contributed to decrease FHB in the susceptible Italian cultivar Marco Aurelio, but the combination of two strategies (resistant genotype × eco-friendly compound) was extremely promising, posing the basis for novel biotechnological approaches forward a sustainable agriculture. More research is needed forward a practical application of chitosan to control FHB, such as studies on production costs and its stability in different environments.

## Supplementary Materials

The Supplementary are available online.

## References

[1] Peng J.H., Sun D., Nevo E. (2011). Domestication evolution, genetics and genomics in wheat. Mol. Breed..

[2] Feldman M., Levy A.A. (2012). Genome evolution due to allopolyploidization in wheat. Genetics.

[3] Charmet G. (2011). Wheat domestication: Lessons for the future. Comptes Rendus Biol..

[4] Dubcovsky J., Dvorak J. (2007). Genome plasticity a key factor in the succes of polyploid wheat under domestication. Science.

[5] Fagnano M., Fiorentino N., D’Egidio M.G., Quaranta F., Ritieni A., Ferracane R., Raimondi G. (2012). Durum wheat in conventional and organic farming: Yield amount and pasta quality in Southern Italy. Sci. World J..

[6] Ma Z., Xie Q., Li G., Jia H., Zhou J., Kong Z., Li N., Yuan Y. (2020). Germplasms, genetics and genomics for better control of disastrous wheat Fusarium head blight. Theor. Appl. Genet..

[7] Khan M.K., Pandey A., Athar T., Choudhary S., Deval R., Gezgin S., Hamurcu M., Topal A., Atmaca E., Santos P.A. (2020). Fusarium head blight in wheat: Contemporary status and molecular approaches. 3 Biotech.

[8] McMullen M., Bergstrom G., De Wolf E., Dill-Macky R., Hershman D., Shaner G., Van Sanford D. (2012). A unified effort to fight an enemy of wheat and barley: Fusarium head blight. Plant Dis..

[9] van der Lee T., Zhang H., van Diepeningen A., Waalwijk C. (2015). Biogeography of *Fusarium graminearum* species complex and chemotypes: A review. Food Addit. Contam. Part A Chem. Anal. Control. Expo. Risk Assess..

[10] Darwish W.S., Ikenaka Y., Nakayama S.M.M., Ishizuka M. (2014). An overview on mycotoxin contamination of foods in Africa. J. Vet. Med. Sci..

[11] Khaledi N., Taheri P., Falahati Rastegar M. (2017). Identification, virulence factors characterization, pathogenicity and aggressiveness analysis of *Fusarium* spp., causing wheat head blight in Iran. Eur. J. Plant Pathol..

[12] Dweba C.C., Figlan S., Shimelis H.A., Motaung T.E., Sydenham S., Mwadzingeni L., Tsilo T.J. (2017). Fusarium head blight of wheat: Pathogenesis and control strategies. Crop Prot..

[13] Bai G., Shaner G. (2004). Management and resistance in wheat and barley to Fusarium head blight. Annu. Rev. Phytopathol..

[14] Parry D.W., Jenkinson P., McLeod L. (1995). Fusarium ear blight (scab) in small grain cereals—A review. Plant Pathol..

[15] Jansen C., von Wettstein D., Schafer W., Kogel K.-H., Felk A., Maier F.J. (2005). Infection patterns in barley and wheat spikes inoculated with wild-type and trichodiene synthase gene disrupted *Fusarium graminearum*. Proc. Natl. Acad. Sci. USA.

[16] Ilgen P., Hadeler B., Maier F.J., Schäfer W. (2009). Developing kernel and rachis node induce the trichothecene pathway of *Fusarium graminearum* during wheat head infection. Mol. Plant Microbe Interact..

[17] Wegulo S.N., Baenziger P.S., Hernandez Nopsa J., Bockus W.W., Hallen-Adams H. (2015). Management of Fusarium head blight of wheat and barley. Crop Prot..

[18] Homdork S., Fehrmann H., Beck R. (2000). Effects of field application of tebuconazole on yield, yield components and the mycotoxin content of *Fusarium*-infected wheat grain. J. Phytopathol..

[19] Mesterházy Á., Bartók T., Lamper C. (2003). Influence of wheat cultivar, species of *Fusarium*, and isolate aggressiveness on the efficacy of fungicides for control of fusarium head blight. Plant Dis..

[20] Champeil A., Fourbet J.F., Doré T., Rossignol L. (2004). Influence of cropping system on Fusarium head blight and mycotoxin levels in winter wheat. Crop Prot..

[21] Simpson D.R., Weston G.E., Turner J.A., Jennings P., Nicholson P. (2001). Differential control of head blight pathogens of wheat by fungicides and consequences for mycotoxin contamination of grain. Eur. J. Plant Pathol..

[22] Pirgozliev S.R., Edwards S.G., Hare M.C., Jenkinson P. (2002). Effect of dose rate of azoxystrobin and metconazole on the development of Fusarium head blight and the accumulation of deoxynivalenol (DON) in wheat grain. Eur. J. Plant Pathol..

[23] Shah L., Ali A., Yahya M., Zhu Y., Wang S., Si H., Rahman H., Ma C. (2018). Integrated control of Fusarium head blight and deoxynivalenol mycotoxin in wheat. Plant Pathol..

[24] Spolti P., Pathology P., Biology P., Ny I. (2014). Triazole sensitivity in a contemporary population of *Fusarium graminearum* from New York wheat and competitiveness of a tebuconazole-resistant isolate. Plant Dis..

[25] Chen Y., Zhou M.G. (2009). Characterization of *Fusarium graminearum* isolates resistant to both carbendazim and a new fungicide JS399-19. Phytopathology.

[26] Nsibande S.A., Forbes P.B.C. (2016). Fluorescence detection of pesticides using quantum dot materials—A review. Anal. Chim. Acta.

[27] Taha S.M., Amer M.E., Elmarsafy A.E., Elkady M.Y. (2014). Adsorption of 15 different pesticides on untreated and phosphoric acid treated biochar and charcoal from water. J. Environ. Chem. Eng..

[28] Margni M., Rossier D., Crettaz P., Jolliet O. (2002). Life cycle impact assessment of pesticides on human health and ecosystems. Agric. Ecosyst. Environ..

[29] Rice P.J., Rice P.J., Arthur E.L., Barefoot A.C. (2007). Advances in pesticide environmental fate and exposure assessments. J. Agric. Food Chem..

[30] Sabarwal A., Kumar K., Singh R.P. (2018). Hazardous effects of chemical pesticides on human health–Cancer and other associated disorders. Environ. Toxicol. Pharmacol..

[31] Mekonen S., Argaw R., Simanesew A., Houbraken M., Senaeve D., Ambelu A., Spanoghe P. (2016). Pesticide residues in drinking water and associated risk to consumers in Ethiopia. Chemosphere.

[32] de Souza R.M., Seibert D., Quesada H.B., de Jesus Bassetti F., Fagundes-Klen M.R., Bergamasco R. (2020). Occurrence, impacts and general aspects of pesticides in surface water: A review. Process Saf. Environ. Prot..

[33] Oerke E.C., Dehne H.W. (2004). Safeguarding production—Losses in major crops and the role of crop protection. Crop Prot..

[34] Shand C., Finck A., Blair G., Tandon H. (2006). Plant nutrition for food security. A guide for integrated nutrient management. Exp. Agric..

[35] Fortunati E., Mazzaglia A., Balestra G.M. (2019). Sustainable control strategies for plant protection and food packaging sectors by natural substances and novel nanotechnological approaches. J. Sci. Food Agric..

[36] Morcia C., Tumino G., Ghizzoni R., Bara A., Salhi N., Terzi V. (2017). In vitro evaluation of sub-lethal concentrations of plant-derived antifungal compounds on FUSARIA growth and mycotoxin production. Molecules.

[37] Gao T., Zhou H., Zhou W., Hu L., Chen J., Shi Z. (2016). The fungicidal activity of thymol against *Fusarium graminearum* via inducing lipid peroxidation and disrupting ergosterol biosynthesis. Molecules.

[38] Dash M., Chiellini F., Ottenbrite R.M., Chiellini E. (2011). Chitosan—A versatile semi-synthetic polymer in biomedical applications. Prog. Polym. Sci..

[39] Sharif R., Mujtaba M., Rahman M.U., Shalmani A., Ahmad H., Anwar T., Tianchan D., Wang X. (2018). The multifunctional role of chitosan in horticultural crops; a review. Molecules.

[40] Kaya M., Akyuz L., Sargin I., Mujtaba M., Salaberria A.M., Labidi J., Cakmak Y.S., Koc B., Baran T., Ceter T. (2017). Incorporation of sporopollenin enhances acid–base durability, hydrophobicity, and mechanical, antifungal and antioxidant properties of chitosan films. J. Ind. Eng. Chem..

[41] Akyuz L., Kaya M., Koc B., Mujtaba M., Ilk S., Labidi J., Salaberria A.M., Cakmak Y.S., Yildiz A. (2017). Diatomite as a novel composite ingredient for chitosan film with enhanced physicochemical properties. Int. J. Biol. Macromol..

[42] Allan C.R., Hadwiger L.A. (1979). The fungicidal effect of chitosan on fungi of varying cell wall composition. Exp. Mycol..

[43] Barber M.S., Bertram R.E., Ride J.P. (1989). Chitin oligosaccharides elicit lignification in wounded wheat leaves. Physiol. Mol. Plant Pathol..

[44] Ni Y., Qian Z., Yin Y., Yuan W., Wu F., Jin T. (2020). Polyvinyl alcohol/chitosan/polyhexamethylene biguanide phase separation system: A potential topical antibacterial formulation with enhanced antimicrobial effect. Molecules.

[45] Mania S., Partyka K., Pilch J., Augustin E., Cieślik M., Ryl J., Jinn J.R., Wang Y.J., Michałowska A., Tylingo R. (2019). Obtaining and characterization of the PLA/chitosan foams with antimicrobial properties achieved by the emulsification combined with the dissolution of chitosan by CO_2_ saturation. Molecules.

[46] Fortunati E., Giovanale G., Luzi F., Mazzaglia A., Kenny J.M., Torre L., Balestra G.M. (2017). Effective postharvest preservation of kiwifruit and romaine lettuce with a chitosan hydrochloride coating. Coatings.

[47] Bhaskara Reddy M.V., Arul J., Ait-Barka E., Angers P., Richard C., Castaigne F. (1998). Effect of chitosan on growth and toxin production by *Alternaria alternata* f. sp. *lycopersici*. Biocontrol Sci. Technol..

[48] Hadwiger L.A. (2013). Multiple effects of chitosan on plant systems: Solid science or hype. Plant Sci..

[49] Lopez-Moya F., Suarez-Fernandez M., Lopez-Llorca L.V. (2019). Molecular mechanisms of chitosan interactions with fungi and plants. Int. J. Mol. Sci..

[50] Maluin F.N., Hussein M.Z. (2020). Chitosan-based agronanochemicals as a sustainable alternative in crop protection. Molecules.

[51] Buzón-Durán L., Martín-Gil J., Marcos-Robles J.L., Fombellida-Villafruela Á., Pérez-Lebeña E., Martín-Ramos P. (2020). Antifungal activity of chitosan oligomers–amino acid aonjugate complexes against *Fusarium culmorum* in spelt (*Triticum spelta* L.). Agronomy.

[52] Alvarez-Carvajal F., Gonzalez-Soto T., Armenta-Calderón A.D., Ibarra R.M., Esquer-Miranda E., Juarez J., Encinas-Basurto D. (2020). Silver nanoparticles coated with chitosan against *Fusarium oxysporum* causing the tomato wilt. Biotecnia.

[53] Lipsa F.D., Ursu E.L., Ursu C., Ulea E., Cazacu A. (2020). Evaluation of the antifungal activity of gold-chitosan and carbon nanoparticles on *Fusarium oxysporum*. Agronomy.

[54] Mejdoub-Trabelsi B., Touihri S., Ammar N., Riahi A., Daami-Remadi M. (2019). Effect of chitosan for the control of potato diseases caused by *Fusarium* species. J. Phytopathol..

[55] Kheiri A., Moosawi Jorf S.A., Mallihipour A., Saremi H., Nikkhah M. (2016). Application of chitosan and chitosan nanoparticles for the control of Fusarium head blight of wheat (*Fusarium graminearum*) in vitro and greenhouse. Int. J. Biol. Macromol..

[56] Kheiri A., Moosawi Jorf S.A., Malihipour A., Saremi H., Nikkhah M. (2017). Synthesis and characterization of chitosan nanoparticles and their effect on Fusarium head blight and oxidative activity in wheat. Int. J. Biol. Macromol..

[57] Gunupuru L.R., Patel J.S., Sumarah M.W., Renaud J.B., Mantin E.G., Prithiviraj B. (2019). A plant biostimulant made from the marine brown algae *Ascophyllum nodosum* and chitosan reduce Fusarium head blight and mycotoxin contamination in wheat. PLoS ONE.

[58] Liang W., Yu A., Wang G., Zheng F., Hu P., Jia J., Xu H. (2018). A novel water-based chitosan-La pesticide nanocarrier enhancing defense responses in rice (*Oryza sativa* L.) growth. Carbohydr. Polym..

[59] Helander I.M., Nurmiaho-Lassila E.L., Ahvenainen R., Rhoades J., Roller S. (2001). Chitosan disrupts the barrier properties of the outer membrane of Gram-negative bacteria. Int. J. Food Microbiol..

[60] Cruz-Romero M.C., Murphy T., Morris M., Cummins E., Kerry J.P. (2013). Antimicrobial activity of chitosan, organic acids and nano-sized solubilisates for potential use in smart antimicrobially-active packaging for potential food applications. Food Control.

[61] Xing K., Zhu X., Peng X., Qin S. (2015). Chitosan antimicrobial and eliciting properties for pest control in agriculture: A review. Agron. Sustain. Dev..

[62] Dong X. (2001). Genetic dissection of systemic acquired resistance. Curr. Opin. Plant Biol..

[63] Gruner K., Griebel T., Návarová H., Attaran E., Zeier J. (2013). Reprogramming of plants during systemic acquired resistance. Front. Plant Sci..

[64] Muthukrishnan S., Liang G.H., Trick H.N., Gill B.S. (2001). Pathogenesis-related proteins and their genes in cereals. Plant Cell. Tissue Organ Cult..

[65] Gao Q.M., Zhu S., Kachroo P., Kachroo A. (2015). Signal regulators of systemic acquired resistance. Front. Plant Sci..

[66] Mackintosh C.A., Lewis J., Radmer L.E., Shin S., Heinen S.J., Smith L.A., Wyckoff M.N., Dill-Macky R., Evans C.K., Kravchenko S. (2007). Overexpression of defense response genes in transgenic wheat enhances resistance to Fusarium head blight. Plant Cell Rep..

[67] Makandar R., Nalam V.J., Lee H., Trick H.N., Dong Y., Shah J. (2012). Salicylic acid regulates basal resistance to Fusarium head blight in wheat. Mol. Plant Microbe Interact..

[68] Qi P.F., Zhang Y.Z., Liu C.H., Chen Q., Guo Z.R., Wang Y., Xu B.J., Jiang Y.F., Zheng T., Gong X. (2019). Functional analysis of FgNahG clarifies the contribution of salicylic acid to wheat (*Triticum aestivum*) resistance against fusarium head blight. Toxins.

[69] Grossmann K., Retzlaff G. (1997). Bioregulatory effects of the fungicidal strobilurin kresoxim-methyl in wheat (*Triticum aestivum*). Pestic. Sci..

[70] Grossmann K., Kwiatkowski J., Caspar G. (1999). Regulation of phytohormone levels, leaf senescence and transpiration by the strobilurin kresoxim-methyl in wheat (*Triticum aestivum*). J. Plant Physiol..

[71] Wang L., Li Q., Liu Z., Surendra A., Pan Y., Li Y., Irina Zaharia L., Ouellet T., Fobert P.R. (2018). Integrated transcriptome and hormone profiling highlight the role of multiple phytohormone pathways in wheat resistance against fusarium head blight. PLoS ONE.

[72] Bönnighausen J., Schauer N., Schäfer W., Bormann J. (2019). Metabolic profiling of wheat rachis node infection by *Fusarium graminearum* – decoding deoxynivalenol-dependent susceptibility. New Phytol..

[73] Daszkowska-Golec A., Szarejko I. (2013). Open or close the gate - Stomata action under the control of phytohormones in drought stress conditions. Front. Plant Sci..

[74] Kang Z., Buchenauer H. (2000). Ultrastructural and immunocytochemical investigation of pathogen development and host responses in resistant and susceptible wheat spikes infected by *Fusarium culmorum*. Physiol. Mol. Plant Pathol..

[75] El Hadrami A., Adam L.R., El Hadrami I., Daayf F. (2010). Chitosan in plant protection. Mar. Drugs.

[76] Katiyar D., Hemantaranjan A., Singh B. (2015). Chitosan as a promising natural compound to enhance potential physiological responses in plant: A review. Indian J. Plant Physiol..

[77] Badawy M.E.I., Rabea E.I. (2016). Chitosan and Its Derivatives as Active Ingredients against Plant Pests and Diseases.

[78] Kumaraswamy R.V., Kumari S., Choudhary R.C., Pal A., Raliya R., Biswas P., Saharan V. (2018). Engineered chitosan based nanomaterials: Bioactivities, mechanisms and perspectives in plant protection and growth. Int. J. Biol. Macromol..

[79] Zeng D., Luo X. (2012). Physiological effects of chitosan coating on wheat growth and activities of protective enzyme with drought tolerance. Open J. Soil Sci..

[80] Ma L., Li Y., Yu C., Wang Y., Li X., Li N., Chen Q., Bu N. (2012). Alleviation of exogenous oligochitosan on wheat seedlings growth under salt stress. Protoplasma.

[81] Edwards S.G., Pirgozliev S.R., Hare M.C., Jenkinson P. (2001). Quantification of trichothecene-producing *Fusarium* species in harvested grain by competitive PCR to determine efficacies of fungicides against fusarium head blight of winter wheat. Appl. Environ. Microbiol..

[82] Zhang Y.J., Fan P.S., Zhang X., Chen C.J., Zhou M.G. (2009). Quantification of *Fusarium graminearum* in harvested grain by real-time polymerase chain reaction to assess efficacies of fungicides on fusarium head blight, deoxynivalenol contamination, and yield of winter wheat. Phytopathology.

[83] Khan M.R., Doohan F.M. (2009). Comparison of the efficacy of chitosan with that of a fluorescent pseudomonad for the control of Fusarium head blight disease of cereals and associated mycotoxin contamination of grain. Biol. Control.

[84] Zachetti V.G.L., Cendoya E., Nichea M.J., Chulze S.N., Ramirez M.L. (2019). Preliminary study on the use of chitosan as an eco-friendly alternative to control *Fusarium* growth and mycotoxin production on maize and wheat. Pathogens.

[85] Ramirez M.L., Chulze S., Magan N. (2004). Impact of environmental factors and fungicides on growth and deoxinivalenol production by *Fusarium graminearum* isolates from Argentinian wheat. Crop Prot..

[86] Blandino M., Minelli L., Reyneri A. (2006). Strategies for the chemical control of Fusarium head blight: Effect on yield, alveographic parameters and deoxynivalenol contamination in winter wheat grain. Eur. J. Agron..

[87] Lemmens M., Scholz U., Berthiller F., Dall’Asta C., Koutnik A., Schuhmacher R., Adam G., Buerstmayr H., Mesterházy Á., Krska R. (2005). The ability to detoxify the mycotoxin deoxynivalenol colocalizes with a major quantitative trait locus for fusarium head blight resistance in wheat. Mol. Plant Microbe Interact..

[88] Su Z., Bernardo A., Tian B., Chen H., Wang S., Ma H., Cai S., Liu D., Zhang D., Li T. (2019). A deletion mutation in TaHRC confers Fhb1 resistance to Fusarium head blight in wheat. Nat. Genet..

[89] Rawat N., Pumphrey M.O., Liu S., Zhang X., Tiwari V.K., Ando K., Trick H.N., Bockus W.W., Akhunov E., Anderson J.A. (2016). Wheat Fhb1 encodes a chimeric lectin with agglutinin domains and a pore-forming toxin-like domain conferring resistance to Fusarium head blight. Nat. Genet..

[90] Pastirčák M., Lemmens M., Šrobárová A. (2002). Reaction of maize hybrids to ear rot caused by *Fusarium graminearum* Schwabe. Plant Prot. Sci..

[91] Prat N., Guilbert C., Prah U., Wachter E., Steiner B., Langin T., Robert O., Buerstmayr H. (2017). QTL mapping of Fusarium head blight resistance in three related durum wheat populations. Theor. Appl. Genet..

[92] Urban M., Daniels S., Mott E., Hammond-Kosack K. (2002). Arabidopsis is susceptible to the cereal ear blight fungal pathogens *Fusarium graminearum* and *Fusarium culmorum*. Plant J..

[93] Granade T.C., Hehmann M.F., Artis W.M. (1985). Monitoring of filamentous fungal growth by in situ microspectrophotometry, fragmented mycelium absorbance density, and 14C incorporation: Alternatives to mycelial dry weight. Appl. Environ. Microbiol..

[94] Ncube N.S., Afolayan A.J., Okoh A.I. (2008). Assessment techniques of antimicrobial properties of natural compounds of plant origin: Current methods and future trends. African J. Biotechnol..

[95] Yun Y., Yin D., Dawood D.H., Liu X., Chen Y., Ma Z. (2014). Functional characterization of FgERG3 and FgERG5 associated with ergosterol biosynthesis, vegetative differentiation and virulence of *Fusarium graminearum*. Fungal Genet. Biol..

[96] Carapito R., Hatsch D., Vorwerk S., Petkovski E., Jeltsch J.M., Phalip V. (2008). Gene expression in *Fusarium graminearum* grown on plant cell wall. Fungal Genet. Biol..

[97] Heidtmann-bemvenuti R., Tralamazza M., Fetter C., Ferreira J., Badiale-furlong E. (2016). Effect of natural compounds on *Fusarium graminearum* complex. Sci. Food Agric..

[98] Horevaj P., Milus E.A., Bluhm B.H. (2011). A real-time qPCR assay to quantify *Fusarium graminearum* biomass in wheat kernels. J. Appl. Microbiol..

[99] Brown D.W., McCormick S.P., Alexander N.J., Proctor R.H., Desjardins A.E. (2001). A genetic and biochemical approach to study trichothecene diversity in *Fusarium sporotrichioides* and *Fusarium graminearum*. Fungal Genet. Biol..

[100] Geiser D.M., Jiménez-Gasco M.D.M., Kang S., Makalowska I., Veeraraghavan N., Ward T.J., Zhang N., Kuldau G.A., O’Donnell K. (2004). FUSARIUM-ID v. 1.0: A DNA sequence database for identifying *Fusarium*. Eur. J. Plant Pathol..

[101] Harris L.J., Balcerzak M., Johnston A., Schneiderman D., Ouellet T. (2016). Host-preferential *Fusarium graminearum* gene expression during infection of wheat, barley, and maize. Fungal Biol..

[102] Lee T., Lee S.H., Shin J.Y., Kim H.K., Yun S.H., Kim H.Y., Lee S., Ryu J.G. (2014). Comparison of trichothecene biosynthetic gene expression between *Fusarium graminearum* and *Fusarium asiaticum*. Plant Pathol. J..

[103] Bustin S.A., Benes V., Garson J.A., Hellemans J., Huggett J., Kubista M., Mueller R., Nolan T., Pfaffl M.W., Shipley G.L. (2009). The MIQE guidelines: Minimum information for publication of quantitative real-time PCR experiments. Clin. Chem..

[104] Leather G.R., Einhellig F.A. (1988). Bioassay of naturally occurring allelochemicals for phytotoxicity. J. Chem. Ecol..

[105] Buerstmayr H., Steiner B., Lemmens M., Ruckenbauer P. (2000). Resistance to fusarium head blight in winter wheat: Heritability and trait associations. Crop Sci..

[106] Buerstmayr H., Lemmens M., Hartl L., Doldi L., Steiner B., Stierschneider M., Ruckenbauer P. (2002). Molecular mapping of QTLs for Fusarium head blight resistance in spring wheat. I. Resistance to fungal spread (type II resistance). Theor. Appl. Genet..

[107] Zadoks J.C., Chang T.T., Konzak C.F. (1974). A decimal code for the growth stages of cereals. Weed Res..

[108] Lu Z.X., Gaudet D., Puchalski B., Despins T., Frick M., Laroche A. (2006). Inducers of resistance reduce common bunt infection in wheat seedlings while differentially regulating defence-gene expression. Physiol. Mol. Plant Pathol..

[109] Mandalà G., Tundo S., Francesconi S., Gevi F., Zolla L., Ceoloni C., D’Ovidio R. (2019). Deoxynivalenol detoxification in transgenic wheat confers resistance to Fusarium head blight and crown rot diseases. Mol. Plant Microbe Interact..

[110] Tenea G.N., Peres Bota A., Cordeiro Raposo F., Maquet A. (2011). Reference genes for gene expression studies in wheat flag leaves grown under different farming conditions. BMC Res. Notes.

[111] Francesconi S., Balestra G.M. (2020). The modulation of stomatal conductance and photosynthetic parameters is involved in Fusarium head blight resistance in wheat. PLoS ONE.

[112] Francesconi S., Mazzaglia A., Balestra G.M. (2019). Different inoculation methods affect components of Fusarium head blight resistance in wheat. Phytopathol. Mediterr..

[113] Sulyok M., Stadler D., Steiner D., Krska R. (2020). Validation of an LC-MS/MS-based dilute-and-shoot approach for the quantification of >500 mycotoxins and other secondary metabolites in food crops: Challenges and solutions. Anal. Bioanal. Chem..

[114] Metsalu T., Vilo J. (2015). ClustVis: A web tool for visualizing clustering of multivariate data using Principal Component Analysis and heatmap. Nucleic Acids Res..

