# Supplementary material for: Chitosan Hydrochloride Decreases Fusarium graminearum Growth and Virulence and Boosts Growth, Development and Systemic Acquired Resistance in Two Durum Wheat Genotypes
Source: Molecules. 2020 Oct 16;25(20):4752. doi: 10.3390/molecules25204752 (PMC7587526; doi:10.3390/molecules25204752)
Supplement: Supplementary file 1 [file molecules-25-04752-s001.pdf]

Supplementary Materials

Table S1 Relative expression levels and standard errors of each *F. graminearum* gene selected and compound (chitosan hydrochloride C, tebuconazole T, azoxystrobin A, and the mixture of tebuconazole and azoxystrobin T + A). Relative expression values were obtained by using the equation $2^{-\Delta\Delta Cq}$ with *TEF, TUB* and *GAPDH* as reference genes and mock treatment used to normalize the relative expression levels. The data represent averages and standard errors for the four independent biological replicates and the four technical replicates examined. The data were subjected to one-way analysis of variance (ANOVA). Different letters among the plant treatments were assessed by means of significant F values by applying the Tukey Honestly Significant Difference Test (Tukey test) at 0.99 confidence level ($p < 0.01$).

**Gene**		**C**	**T**	**A**	**T + A**
	Relative expression	0.01	2.19	2.58	0.92
Erg3	Standard error (±)	0.044	0.065	0.047	0.05
	$p < 0.01$	b	a	a	ab
	Relative expression	0.33	0.59	0.61	0.87
Fg03624	Standard error (±)	0.02	0.12	0.13	0.04
	$p < 0.01$	b	ab	ab	a
	Relative expression	0.01	0.38	0.11	0.12
Fg03695	Standard error (±)	0.06	0.38	0.01	0.09
	$p < 0.01$	c	a	bc	b
	Relative expression	0.01	0.15	0.50	0.23
Fg07551	Standard error (±)	0.07	0.10	0.13	0.01
	$p < 0.01$	b	b	a	ab
	Relative expression	0.04	1.54	0.36	1.18
Fg09443	Standard error (±)	0.06	0.06	0.18	0.11
	$p < 0.01$	c	a	bc	ab
	Relative expression	0.01	0.57	0.08	0.88
Fg03725	Standard error (±)	0.01	0.12	0.08	0.07
	$p < 0.01$	c	ab	bc	a
	Relative expression	0.04	9.64	2.69	5.69
Fg01572	Standard error (±)	0.05	0.07	0.03	0.03
	$p < 0.01$	c	a	bc	ab
	Relative expression	0.01	1.30	0.64	1.58
Fg04196	Standard error (±)	0.02	0.03	0.14	0.09
	$p < 0.01$	b	a	ab	a
	Relative expression	0.04	1.23	2.44	0.93
Fg06619	Standard error (±)	0.08	0.06	0.16	0.08
	$p < 0.01$	b	ab	a	ab
Fg10231	Relative expression	0.10	4.00	4.90	4.30
	Standard error (±)	0.01	0.03	0.03	0.11

	$p < 0.01$	b	a	a	a
FPP	Relative expression	0.11	3.25	1.33	4.38
	Standard error (±)	0.01	0.77	0.22	0.22
	$p < 0.01$	c	ab	bc	a
Tri1	Relative expression	0.02	0.03	1.30	0.31
	Standard error (±)	0.07	0.10	0.09	0.09
	$p < 0.01$	b	b	a	b
Tri3	Relative expression	0.01	0.82	1.33	0.30
	Standard error (±)	0.04	0.15	0.23	0.08
	$p < 0.01$	c	ab	a	bc
Tri4	Relative expression	0.02	0.07	0.21	0.03
	Standard error (±)	0.00	0.18	0.03	0.01
	$p < 0.01$	b	b	a	b
Tri5	Relative expression	0.02	1.67	2.76	0.60
	Standard error (±)	0.07	0.15	0.34	0.33
	$p < 0.01$	b	ab	a	b
Tri6	Relative expression	0.03	2.09	2.05	0.82
	Standard error (±)	0.08	0.39	0.16	0.16
	$p < 0.01$	c	a	ab	bc
Tri8	Relative expression	3.34	19.94	11.52	16.81
	Standard error (±)	0.09	5.23	2.29	2.94
	$p < 0.01$	b	a	ab	a
Tri9	Relative expression	0.01	4.98	2.04	0.52
	Standard error (±)	0.00	0.01	0.58	0.13
	$p < 0.01$	c	a	bc	bc
Tri10	Relative expression	0.02	2.73	4.19	0.18
	Standard error (±)	0.01	0.84	0.72	0.04
	$p < 0.01$	b	ab	a	b
Tri11	Relative expression	0.15	26.84	4.90	1.44
	Standard error (±)	0.01	0.92	0.76	0.55
	$p < 0.01$	c	a	b	c
Tri12	Relative expression	0.05	24.95	13.21	5.12
	Standard error (±)	0.03	0.37	1.95	0.76
	$p < 0.01$	c	a	b	c
Tri14	Relative expression	0.10	3.39	10.62	2.90
	Standard error (±)	0.01	0.59	1.86	1.01

	$p < 0.01$	b	b	a	b
Tri101	Relative expression	5.00	24.31	0.16	31.40
	Standard error (±)	1.76	3.01	0.01	0.65
	$p < 0.01$	c	b	c	a

Table S2 Relative expression levels and standard errors of each wheat gene selected for each durum wheat genotype (Marco Aurelio and DBC480) and compound (chitosan C, tebuconazole T, azoxystrobin A, and the mixture of tebuconazole and azoxystrobin T + A). Fg represents the *F. graminearum* inoculated control. Relative expression values were obtained by using the equation $2^{-\Delta\Delta Cq}$ with *TaFNR, TaTUB* and *TaACT* as reference genes and mock treatment used to normalize the relative expression levels. The data represent averages and standard errors for the four independent biological replicates and the four technical replicates examined. The data were subjected to two-way analysis of variance (ANOVA). Different letters among the plant treatments were assessed by means of significant F values by applying the Tukey Honestly Significant Difference Test (Tukey test) at 0.99 confidence level ($p < 0.01$).

		**Marco Aurelio**					**DBC480**				
**Gene**		**Fg**	**C**	**T**	**A**	**T + A**	**Fg**	**C**	**T**	**A**	**T + A**
TaPAL	Relative expression	2.89	1.76	1.16	1.09	0.84	2.62	2.35	0.67	2.29	0.27
	Standard error (±)	0.22	0.29	0.22	0.27	0.23	0.84	0.66	0.10	0.80	0.07
	$p < 0.01$	a	b	b	b	c	a	a	c	a	c
TaPR1	Relative expression	3.48	2.91	0.87	0.98	1.05	2.04	2.16	1.21	1.47	0.84
	Standard error (±)	0.27	0.61	0.22	0.18	0.28	0.56	0.59	0.16	0.44	0.12
	$p < 0.01$	a	b	c	c	c	b	b	c	bc	c
TaPR2	Relative expression	2.51	2.69	0.47	0.49	0.40	3.41	3.23	1.79	1.04	0.61
	Standard error (±)	0.69	0.34	0.16	0.13	0.05	0.52	0.83	0.26	0.20	0.08
	$p < 0.01$	b	b	d	d	d	a	a	b	c	d

Table S3 A list of *F. graminearum* selected genes, accession numbers, gene functions, primer pairs, amplicon length from DNA and cDNA and primer pairs references.

**Gene and Accession Number**	**Function**	**Primer Pairs (5'-3')**	**bp (DNA)**	**bp (cDNA)**	**Reference**
Erg3 - XM_011320132.1	C5-sterol desaturase	ACCAACAACCCTGTCGTCAA<br>TCCGTATAAGTGCGGTTGTCA	239	239	Yun et al., 2014
Fg03624 - XM_011323775.1	Xylanase	CTTCCGGTGCTCAGAAGAAGG<br>CGGACCAGTACTGCTGGAAGG	87	87	Carapito et al., 2008
Fg03695 - XM_011323698.1	Endoglucanase	GCATCAACCTCAAGGTCACTGG<br>TGCGTAGAGGTTGAAGAGAATGC	127	127	Carapito et al., 2008
Fg07551 - XM_011329028.1	Exopolygalacturonase	GGCTCTCTTGGCCAGTACAAGG<br>AAGGTGACGTTGTCGATGAAGC	126	126	Carapito et al., 2008
Fg09443 - XM_011329987.1	Pectin methyl esterase	TGTTGAGGAGAAGACCACCTTCC<br>AGAAGAGCCACCGTTGCTACC	121	121	Carapito et al., 2008

Fg03725 - XM_011323666.1	Multidrug transporter	TGGTCGTCAATACCTCTTCATTGG<br>TACCACCAGCGTTGGTAAGAGG	111	111	Carapito et al., 2008
Fg01572 - XM_011319083.1	Glutamate decarboxylase	GGCCAATACTACCAGCTCATCC<br>CGAACGTTCTGACATGATGACG	115	115	Carapito et al., 2008
Fg04196 - XM_011323131.1	Succinate semialdehyde dehydrogenase	GCCGGATACTTCTTCTCCAAGG<br>CGTAACCGCTCTCCTTGACACC	126	126	Carapito et al., 2008
Fg06619 - XM_011327936.1	NADP-dependent alcohol dehydrogenase	CTCAGCCTCCTCGATATTCACG<br>AAGTGAGACGTGCCGATGTAGC	122	122	Carapito et al., 2008
Fg10231 - XM_011320878.1	NADH-ubiquinone oxidoreductase	CCAAGGCCACTGCTACTGATCC<br>CAGATCCTTCCTTGTGGTCTCG	115	115	Carapito et al., 2008
FPP - XM_011328132.1	Farnesyl pyrophosphate synthase	TTTGGCAAGCCCGAACACATT<br>GCGGATCTGGCCAACAACCTTCT	234	234	Lee et al., 2014
Tri1 - AY339129.1	Cytochrome P450 monoxygenase	GCTCGCGAACTAATCACTCC<br>TCCAAACATTGGCAGATGA	185	185	Heidtmann-Bemvenuti et al., 2015
Tri3 - XM_009265280.1	Trichothecene 15-*O*-acetyltransferase	CTTGCAGGGATATCAAGAAATGTTACGA<br>CTCGCCTGTTGTAGTTCGCTTGATTT	208	208	Lee et al., 2014
Tri4 - EF685280.1	Trichodiene oxygenase	TCGAGGCACAACAGAAGGGTATCC<br>AATGTCGGCCTTGGTGGTGTC	237	237	Lee et al., 2014
Tri5 - XM_011323870.1	Trichodiene synthase	GGAGCGTATCGA GAATTTGC<br>TGCCCAACTGTATACGACCA	168	168	Heidtmann-Bemvenuti et al., 2015
Tri6 - AB017495.1	Trichothecene transcriptional regulator	TCTTTGTGAGCGGACGGGACTTTA<br>ATCTCGCATGTTATCCACCCTGCT	245	245	Horevaj et al., 2011
Tri8 - HG970333.1	Trichothecene 3-*O*-esterase	GCTACTTTGGACTCAATTCG<br>CATACTGTACCGCAAGTTCTG	139	139	Lee et al., 2014
Tri9 - LT222054.1	Unknown	AGCCGCTAAACTGATCGACTCATA<br>GCTTTGGCTGCGACCCATAT	85	85	Lee et al., 2014
Tri10 - AF365969.1	Regulatory gene	GTGGCCGGGACGCTTCAAT<br>ATCCGTCAAGTCTTCCCATCTCAT	254	254	Brown et al., 2001
Tri11 - XM_009265275.1	Trichothecene *C*-15 hydroxylase	AAGTACTTCACCCGACCAAACGAC<br>CGGCAAGGCGAATGTCAAAC	192	192	Lee et al., 2014
Tri12 - XM_009265274.1	Trichothecene efflux pump	TCCACAGTCATCTTTCCCCAGTCT<br>CTCCCAGTGCCATAGCGAAGTAGT	172	172	Lee et al., 2014
Tri14 - XM_009265272.1	Unknown	CTGGGAACCTACGCATCAAACATT<br>CGAATGAGCTGCCCAATGATGT	165	165	Lee et al., 2014
Tri101 - AB011417.1	Trichothecene 3-*O*-acetyltransferase	GTGGGACTCTGGGATTACGACTTT<br>GTCCACTCCTTATCCGCCTTCAA	130	130	Lee et al., 2014

TEF - MH572267.1	Translational elongation factor 1-α	ATGGGTAAGGAGGACAAGAC<br>GGAAGTACCAGTGATCATGTT	700	324	Geiser et al., 2004
TUB - XM_011329885	β-tubulin	TTGCATTGGTACACTGGTGAGG<br>AGGCAGCTCCTCCTCGTACTCC	110	110	Carapito et al., 2008
GAPDH - XM_011326605.1	Glyceraldehyde-3-phospate dehydrogenase	TGACTTGACTGTTCGCCTCGAGAA<br>ATGGAGGAGTTGGTGTTGCCGTTA	160	160	Harris et al., 2016

Table S4 A list of wheat selected genes, accession numbers, gene functions, primer pairs, amplicon length from DNA and cDNA and primer pairs references.

Gene and Accession Number	Function	Primer Pairs (5'-3')	bp (DNA)	bp (cDNA)	Reference
TaPAL - X99705	Phenylalanine ammonia-lyase	CGATGCTCGTCCGAGTCAAT<br>CATGACCTCACAGAAGACGC	407	407	*De novo* designed
TaPR1 - AJ007348	Pathogenesis related protein-1	ACTACGACTACGGGTCCAACA<br>TCGTAGTTGCAGGTGATGAAG	145	145	Lu et al., 2006
TaPR2 - Y18212	β-1,3-glucanase	AACGTGCGCCCCTACTACC<br>GCGTCGAACAGGCTCGTGTA	398	398	Francesconi et al., 2020
TaFNR - AJ457980	Ferredoxin-NADP(H)-oxidoreductase	CACCGGCCCAGTGATCTT<br>AAGGGCGTCTGCTCCAACT	259	69	Tenea et al., 2011
TaTUB - *TAU76745*	β-tubulin	CGAGGAGGGCGAGTACGA<br>AGCAAAGCACGACATGGACAT	79	79	Tenea et al., 2011
TaACT - AB181991	Actin	TCCTGTGTTGCTGACTGAGG<br>GGTCCAAACGAAGGATAGCA	350	236	Mandalà et al., 2019

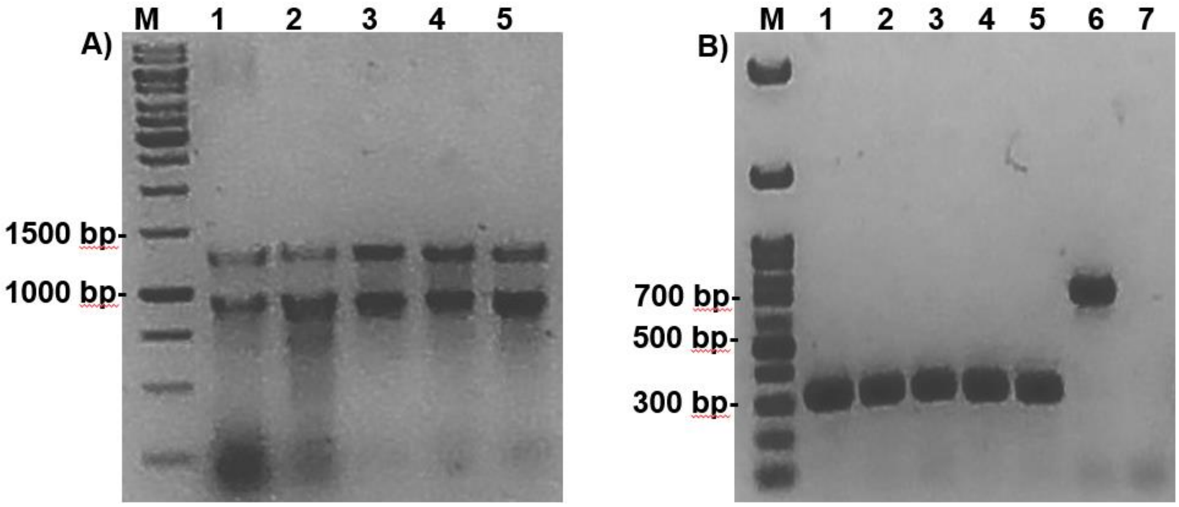

Figure S1 1.5% agarose gel of total extracted RNA (A) and RT-PCR (B) from *F. graminearum.* M) GeneRuler™ 1 kb DNA Ladder (Thermo Fisher Scientific) and ExcelBand™ 100 bp + 3K DNA Ladder (Smobio); (1) Chitosan hydrochloride; (2) Tebuconazole; (3) Azoxystrobin; (4) Tebuconazole+Azoxystrobin; (5) Mock; (6) Genomic DNA (gDNA); (7) No template control (NTC). The figure represents the original pictures of the gels.

Figure S2 Durum seedlings at 14 days after the coating with chitosan hydrochloride at 0.5% (C). M represents the mock control.

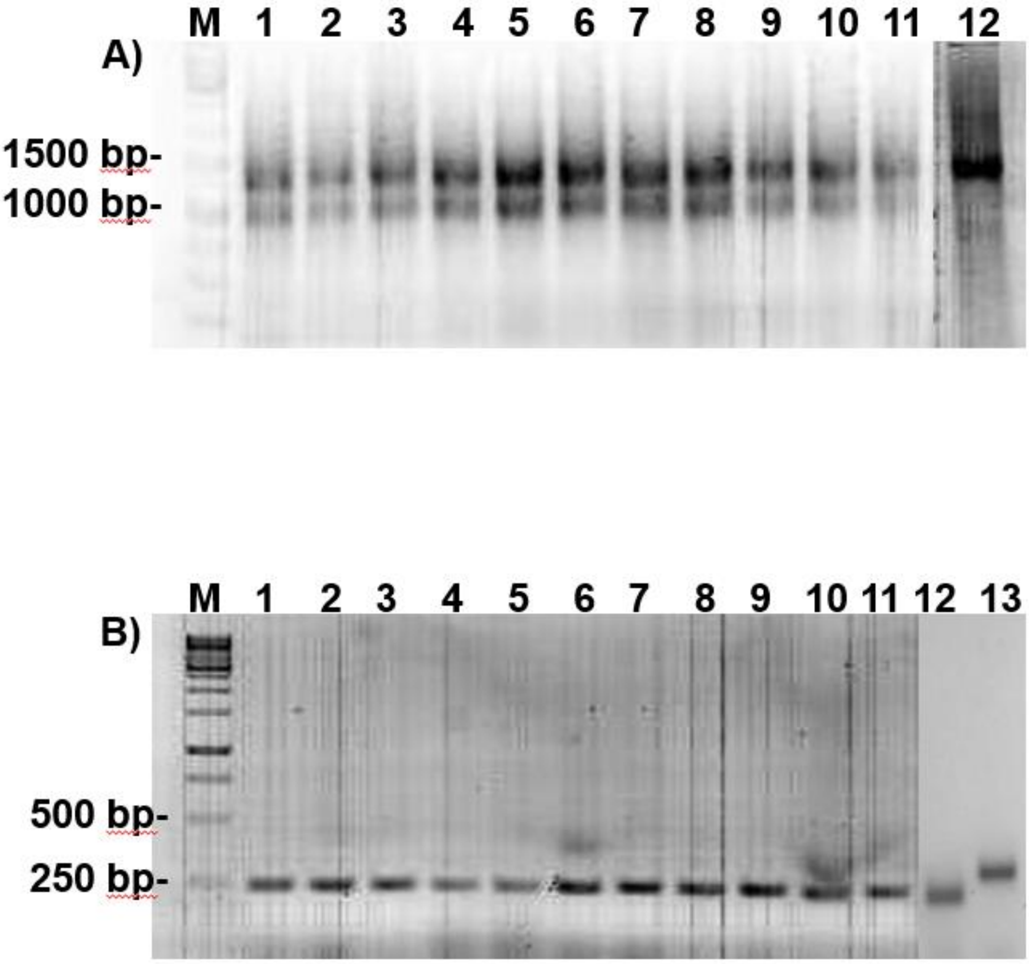

Figure S3 1.5% agarose gel of total extracted RNA (A) and RT-PCR (B) from the spikes of Marco Aurelio and DBC480 subjected to the different compounds' treatments at the flag leaf level. M) GeneRuler™ 1 kb DNA Ladder (Thermo Fisher Scientific); (1) Marco Aurelio, chitosan; (2) Marco Aurelio, tebuconazole; (3) Marco Aurelio, azoxystrobin; (4) Marco Aurelio, tebuconazole+azoxystrobin; (5) Marco Aurelio, *F. graminearum* inoculated control; (6) Marco Aurelio, mock control; (7) DBC480, chitosan; (8) DBC480, tebuconazole; (9) DBC480, azoxystrobin; (10) DBC480, tebuconazole+azoxystrobin; (11) DBC480, *F. graminearum* inoculated control; (12) DBC480, mock control; (13) Genomic DNA (gDNA). The figure represents the pictures of three gels. The original pictures of the gels are available if requested.
